# Supplementary figures and images for: Inhibitors of ApiAP2 protein DNA binding exhibit multistage activity against Plasmodium parasites
Source: PLoS Pathog. 2022 Oct 12;18(10):e1010887. doi: 10.1371/journal.ppat.1010887 (PMC9591056; doi:10.1371/journal.ppat.1010887)

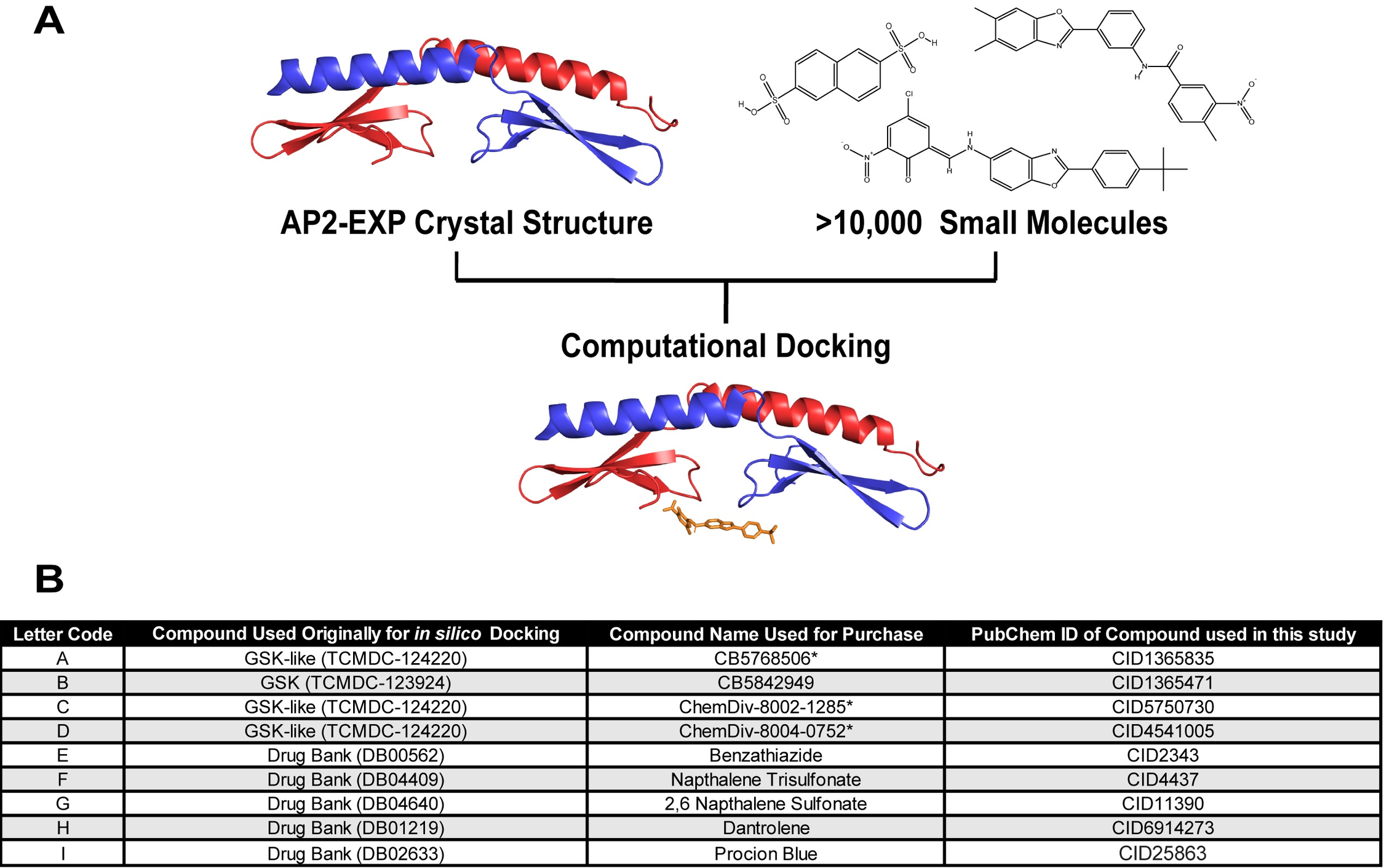

Supplement: S1 Fig — A) The crystal structure of AP2-EXP (PDB:3IGM) [40] was used as a template to computationally dock thousands of small molecules in silico using AutoDock. Results were filtered for compounds that dock within 10 Angstroms of DNA binding residues with a free energy less than -5kJ/mol. Compounds matching these criteria were sourced and used for further testing. B) Seven compounds were identified as putative ApiAP2 competitors in an in-silico screen (Column 2). Six of these were available for direct purchase (Column 3). For the remaining compound (TCMDC-124220), three alternate choices with a Tanimoto similarity score of .9 or greater were purchased (denoted by an asterisk in Column 3). The PubChem ID used to purchase each compound in this study is listed in Column 4. (TIF) [file ppat.1010887.s012.tif]

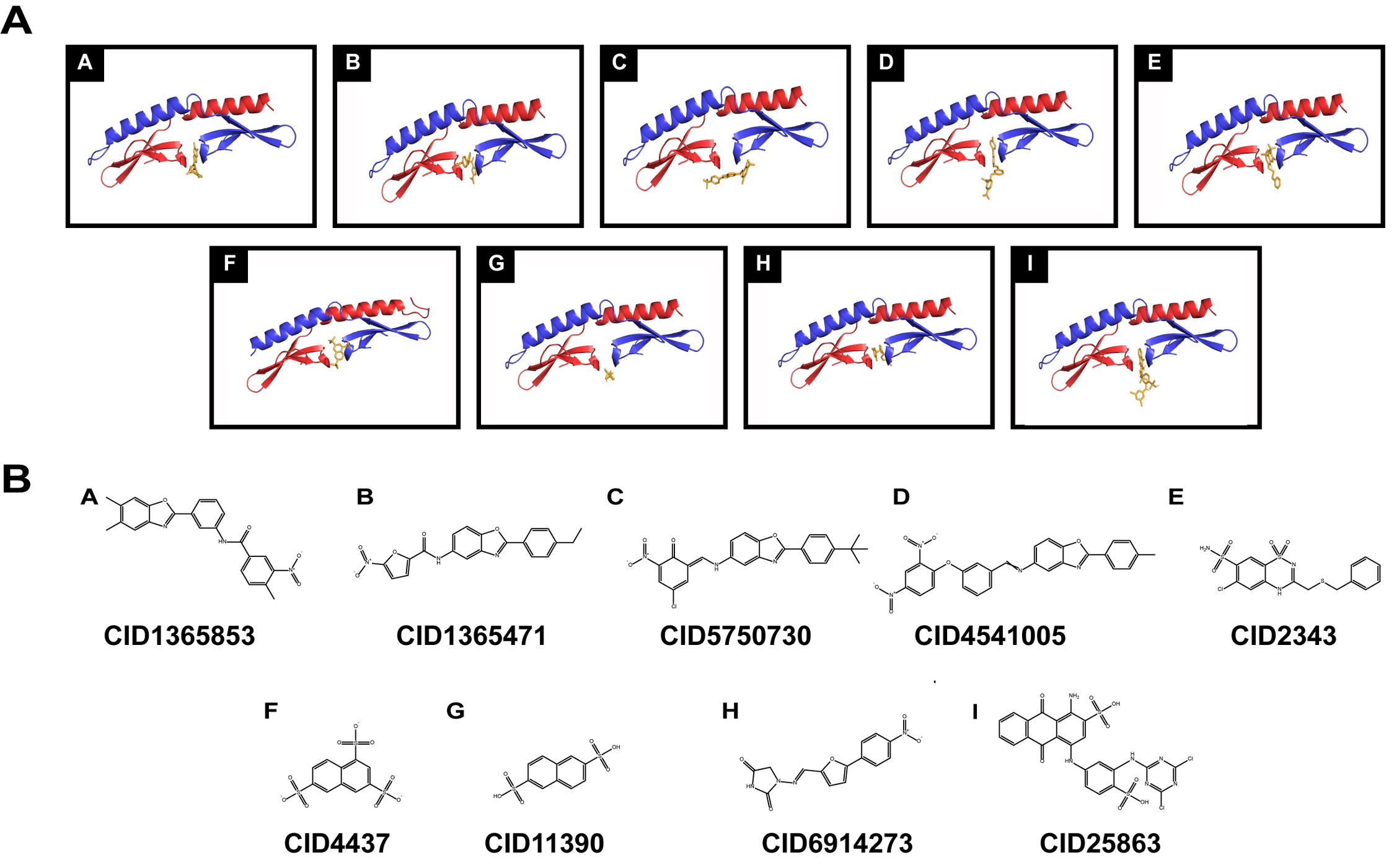

Supplement: S2 Fig — A) The spatial conformation of each of the nine compounds (A-I) that dock within 10 Angstroms of the DNA binding pocket of AP2-EXP with a free energy less than -5kJ/mol is depicted above. B) Chemical structures of each compound (A-I) corresponding to the molecular docking results in panel A. (TIF) [file ppat.1010887.s013.tif]

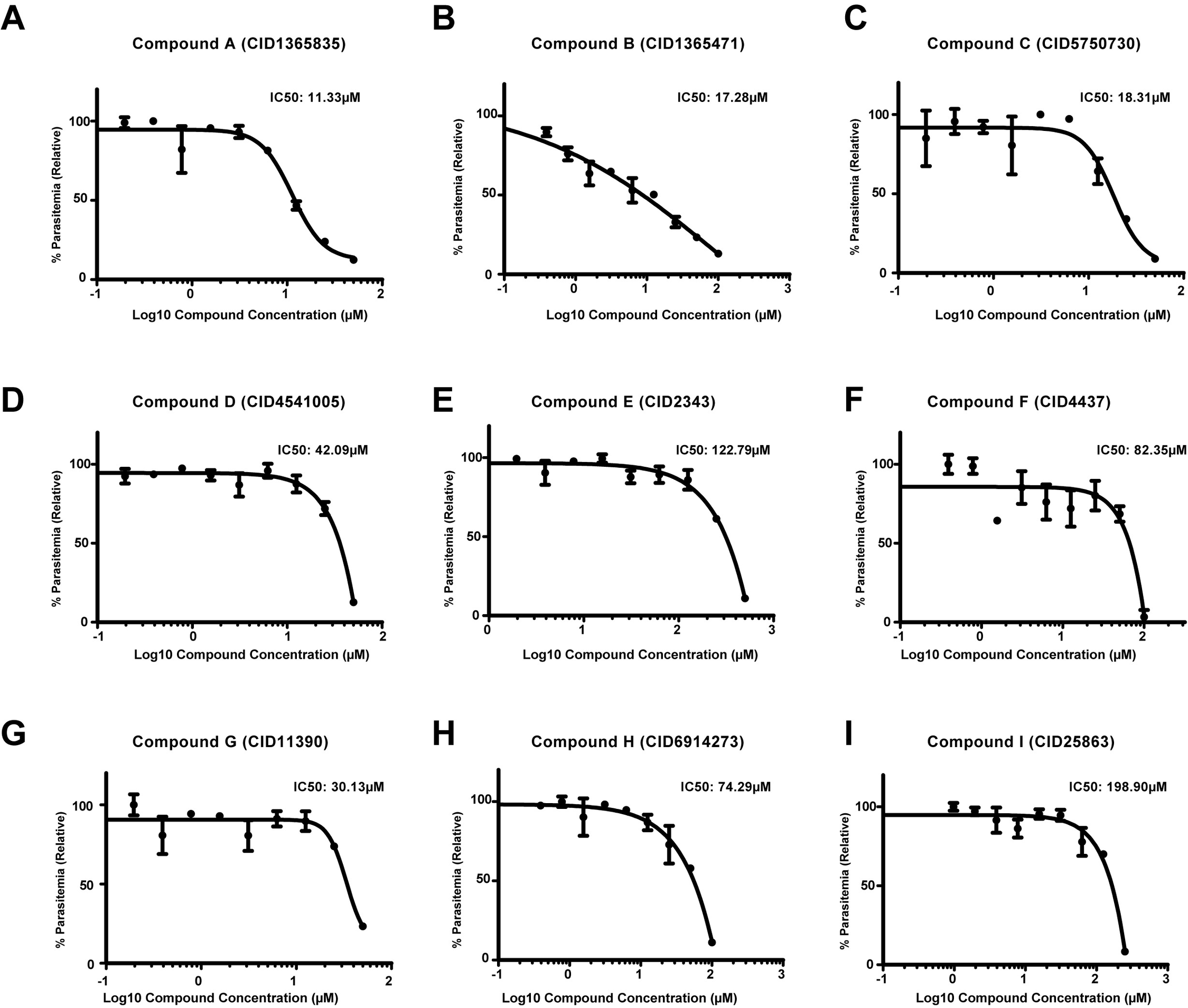

Supplement: S3 Fig — A-I) 48-hour Sybr Green growth inhibition assays were conducted for each of the nine putative ApiAP2 competitor compounds in order to determine IC50 values against asexual P. falciparum. All growth assays were performed in triplicate. All compounds kill asexual stage P. falciparum parasites in the micromolar (11.33–198.90μM) concentration range. Error bars represent standard deviation of the mean. (TIF) [file ppat.1010887.s014.tif]

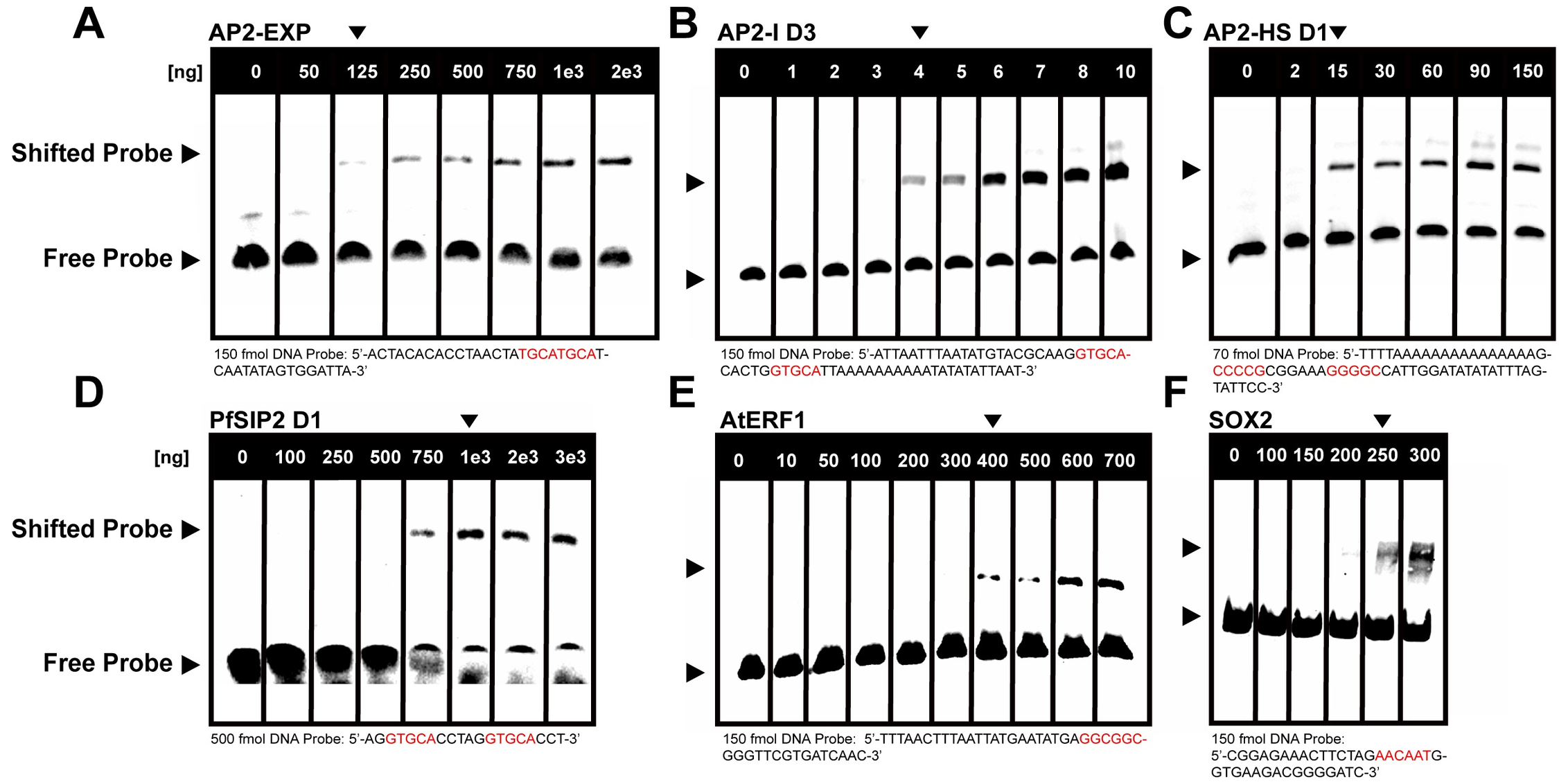

Supplement: S4 Fig — A-F) DNA binding domains AP2-EXP, AP2-I D3, AP2-HS D1, PfSIP2 D1, AtERF1, and full length SOX2 were titrated against DNA oligos containing their respective binding motifs (highlighted in red) in an EMSA. Unless otherwise specified, the minimum mass of each recombinant DNA binding domain required to visualize DNA binding (denoted by an arrow) was used in competitive EMSAs with putative ApiAP2 competitor compounds. (TIF) [file ppat.1010887.s015.tif]

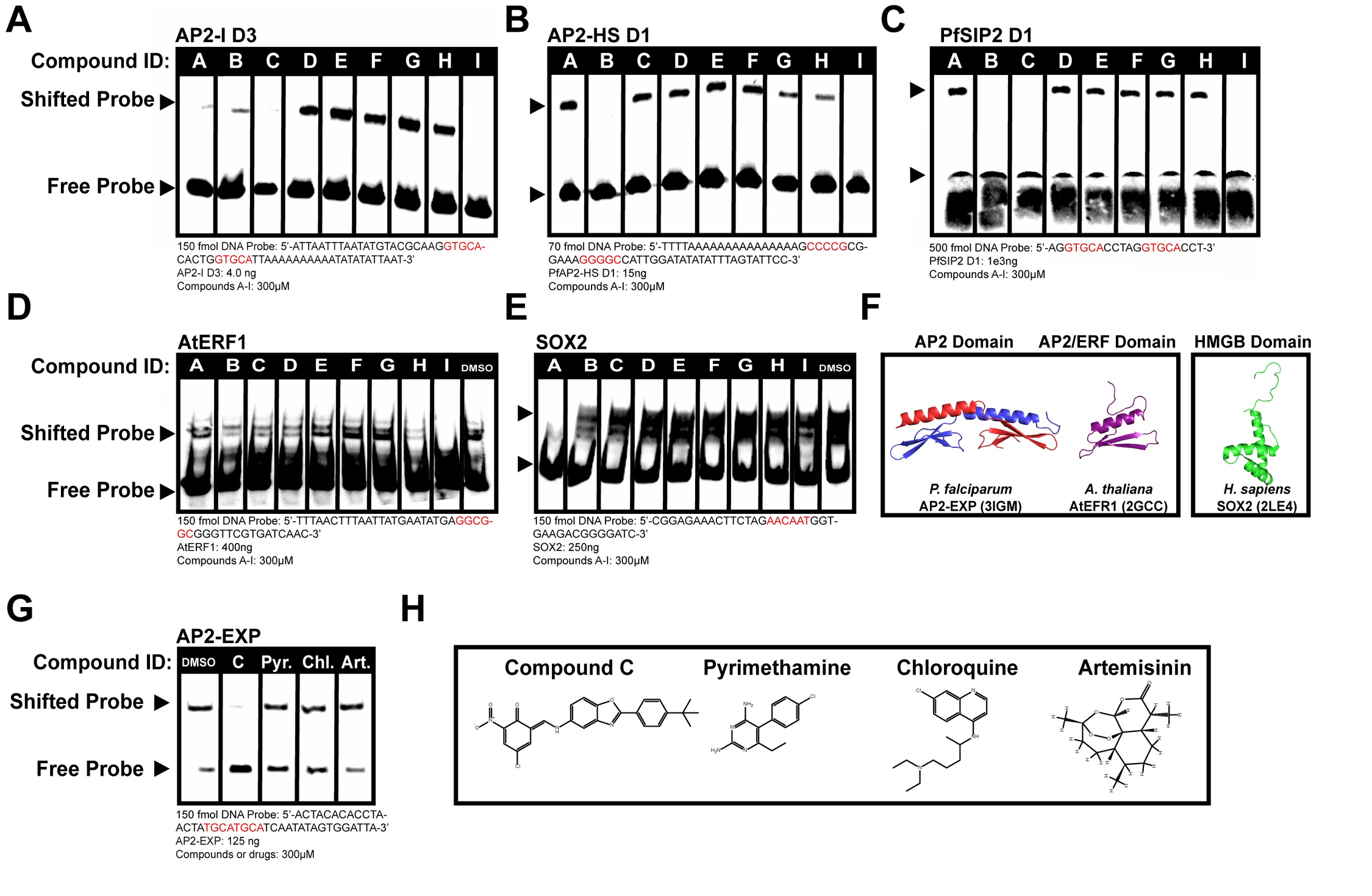

Supplement: S5 Fig — A-C) AP2-I D3 (A) DNA binding activity is competed by Compounds A, B, C and I. AP2-HS D1 (B) is competed by Compounds B and I, and PfSIP2 D1 (C) is competed by Compounds B, C, and I. Compounds A, B, C and I all compete at least one AP2 domain in addition to AP2-EXP. Cognate DNA motifs for each protein are highlighted in red. 300μM of each compound was used per lane. D) The plant encoded Arabidopsis thaliana AP2 domain from Ethylene Response Factor 1 (AtERF1) is competed by Compound I. The Plasmodium AP2 domain competitors Compound A, B, and C do not compete AtERF1. The cognate AtERF1 DNA motif is highlighted in red. 300μM of each compound was used per lane. E) The human encoded High Mobility Group Box Domain transcription factor SOX2 is competed by Compound A. Due to the lack of homology between SOX2 and AP2 domain proteins, this result indicates that Compound A’s DNA binding competition activity is not unique to the AP2 domain. The cognate SOX2 DNA motif is highlighted in red. 300μM of each compound was used per lane. F) The three-dimensional structures of AP2-EXP [40] (PDB:3IGM), AtERF1 [44] (PDB: 2GCC), and SOX2 [46] (PDB: 2LE4). AP2-EXP and AtERF1 bind DNA via contacts with the beta strands [40], while SOX2 binds DNA via contacts with its alpha helices [96]. G) 300μM of the antimalarial compounds pyrimethamine, chloroquine, or artemisinin, were added to a competitive EMSA with AP2-EXP. Compound C was used as a control for activity against AP2-EXP. DMSO was used as a vehicle control. The AP2-EXP cognate DNA motif is highlighted in red. H) The chemical structures of Compound C, pyrimethamine, chloroquine, and artemisinin. (TIF) [file ppat.1010887.s016.tif]

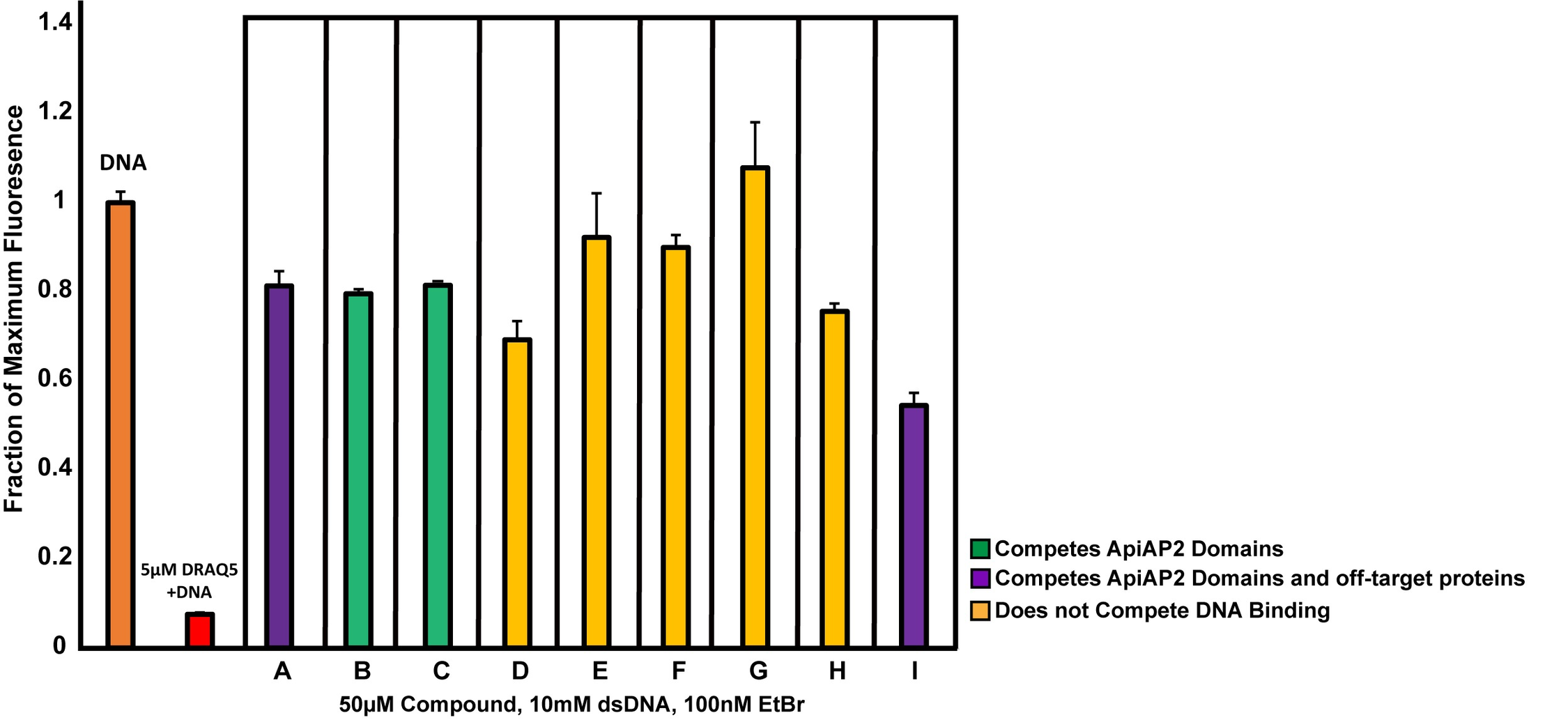

Supplement: S6 Fig — Each putative ApiAP2 competitor compound was added into a mixture containing double stranded DNA and ethidium bromide. The positive control DNA major groove intercalator DRAQ5 knocks down ethidium bromide fluorescence nearly completely relative to the DNA and ethidium bromide control. The legend indicates the cumulative result for each compound in competitive EMSAs. Each assay was performed in triplicate. Error bars represent standard deviation of the mean. (TIF) [file ppat.1010887.s017.tif]

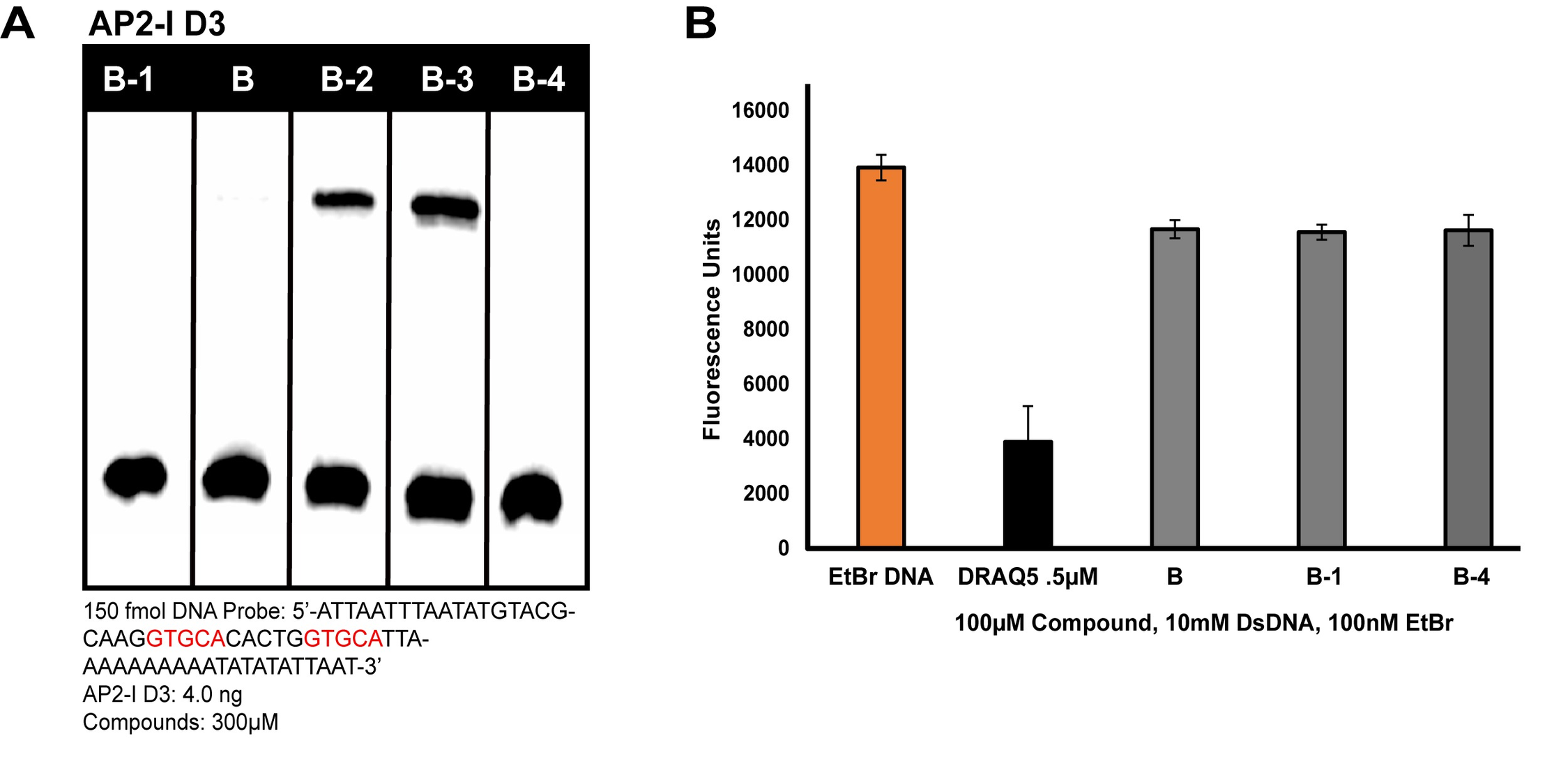

Supplement: S7 Fig — A) Compounds B, B-1, B-2, B-3, and B-4 were added to an EMSA with AP2-I D3 to check whether their DNA binding competition is consistent with AP2-EXP. The cognate AP2-I D3 DNA motif is highlighted in red. 300μM of each compound was used per lane. B) Compounds B, B-1, B-2, B-3, and B-4 were tested for DNA major groove intercalation in an ethidium bromide exclusion assay. DRAQ5 was used as a positive control for intercalation. Each assay was performed in triplicate. Error bars represent standard deviation of the mean. (TIF) [file ppat.1010887.s018.tif]

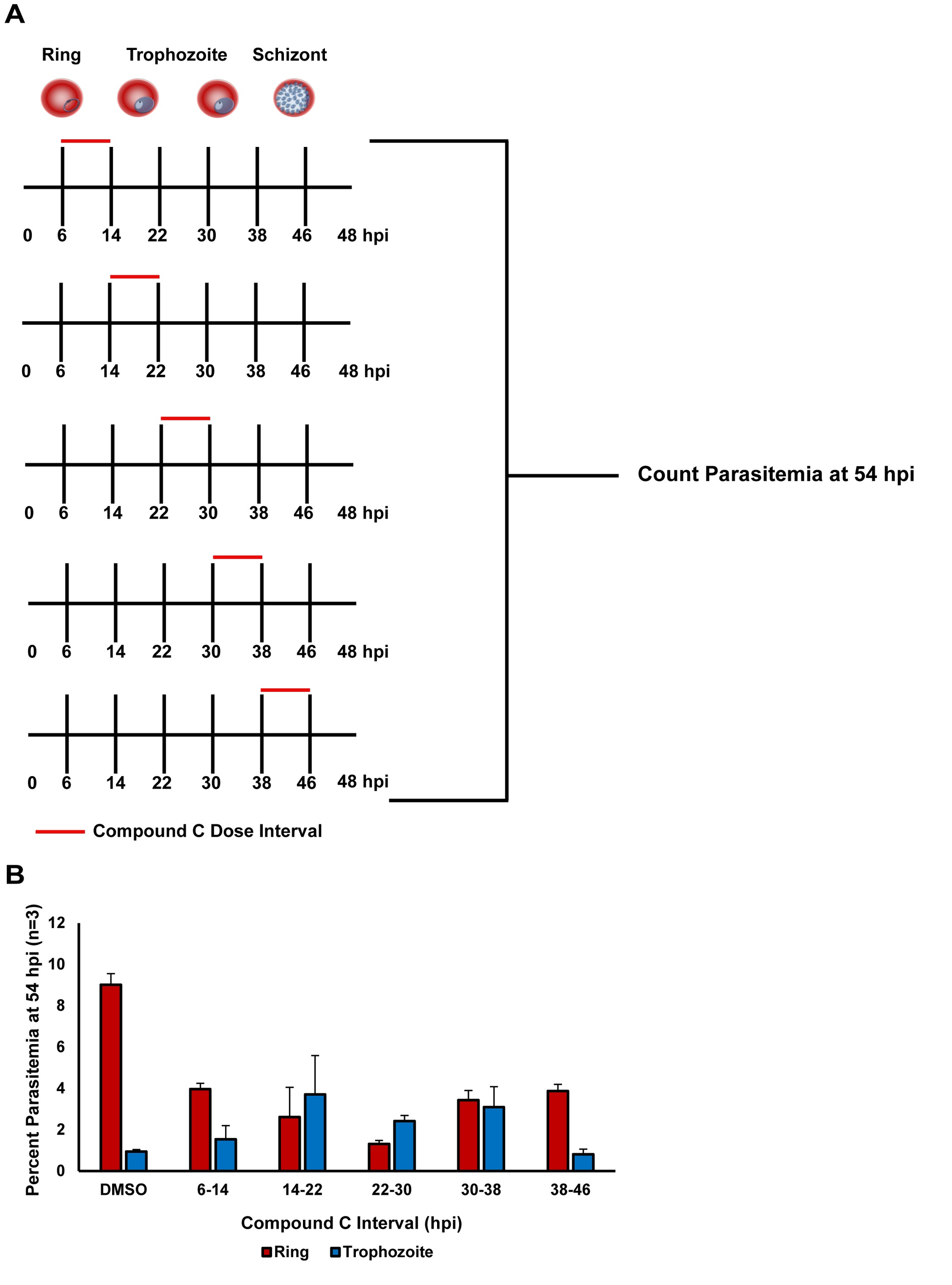

Supplement: S8 Fig — A) Schematic depicting the phenotyping time course. 40μM Compound C was added to wild type Pf3D7 parasite cultures at 1% starting parasitemia for fixed 8-hour intervals starting at 6 hpi throughout the IDC. The interval of Compound C dosage is indicated by each red bar. B) At 54 hpi following Compound C dose intervals, the parasitemia and morphology of each culture was counted. The total percentage of reinvaded rings and stalled trophozoites in each culture was recorded. The ratio of rings to trophozoites is reported in Fig 3F. Error bars represent standard error of the mean. The assay was performed in biological and technical triplicate. (TIF) [file ppat.1010887.s019.tif]

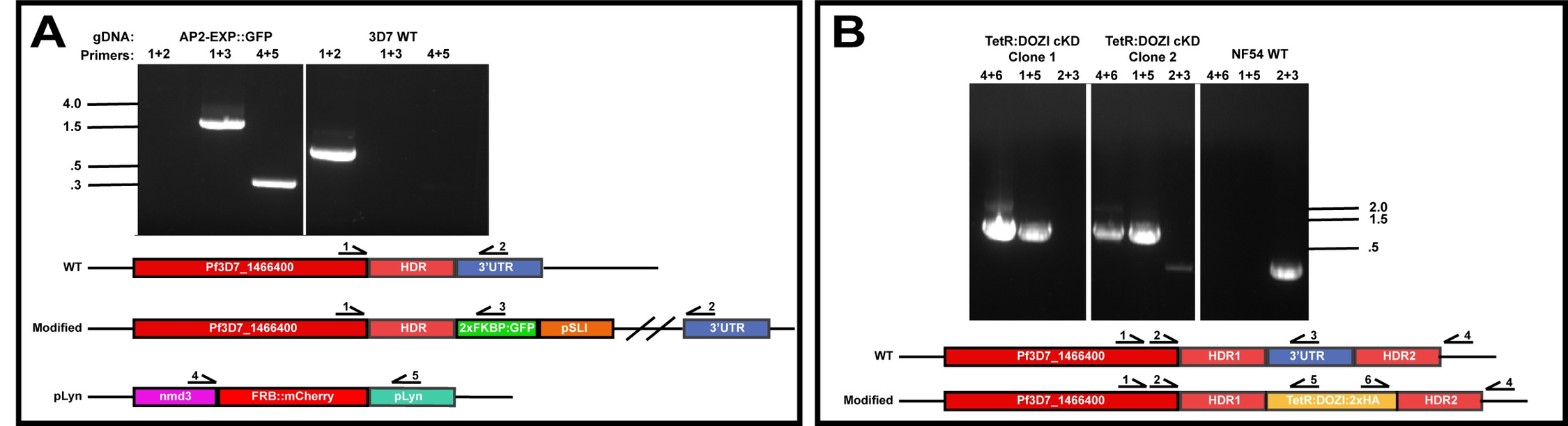

Supplement: S9 Fig — A) The Selection Linked Integration system was used to add 2xFKBP inducible mislocalization protein and GFP to AP2-EXP by single homologous recombination. Successful integration was confirmed by genotyping PCR. Genomic DNA from the wild type Pf3D7 parental line was used as a control. In order to test the efficacy of the knock sideways system, the pLyn mislocalizer plasmid was added to AP2-EXP::GFP and confirmed by PCR. DNA kb are indicated by the marks to the left of the gel. B) The PSN054 TetR:DOZI plasmid was used to add the TetR:DOZI mRNA repression module and endogenous 2xHA tag to AP2-EXP by double homologous recombination. Correct integration was confirmed by genotyping PCR. The parental NF54 parasite line was used as the unedited control. Clonal populations one and two are indicated as C1 and C2, respectively. Clone one had correct integration and complete absence of the wild type ap2-exp DNA locus and was used for further experiments. DNA kb are indicated by the marks to the right of the gel. (TIF) [file ppat.1010887.s020.tif]

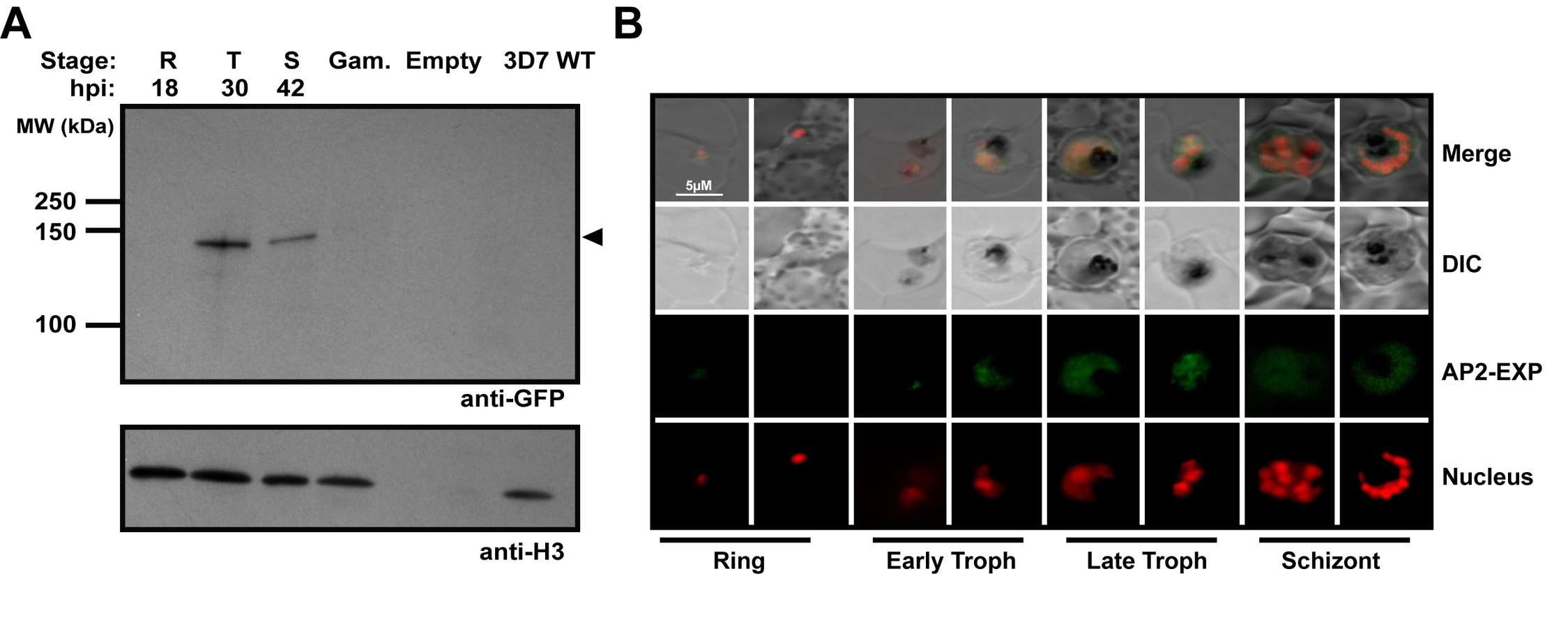

Supplement: S10 Fig — A) AP2-EXP expression was tracked throughout the IDC by harvesting protein from highly synchronous asexual blood stage parasites followed by a western blot against the GFP tag. Histone H3 was used as a loading control. B) AP2-EXP::GFP expression was monitored in a highly synchronous parasite population by fluorescent microscopy across the IDC. DRAQ5 was used as a nuclear stain for parasites. (TIF) [file ppat.1010887.s021.tif]

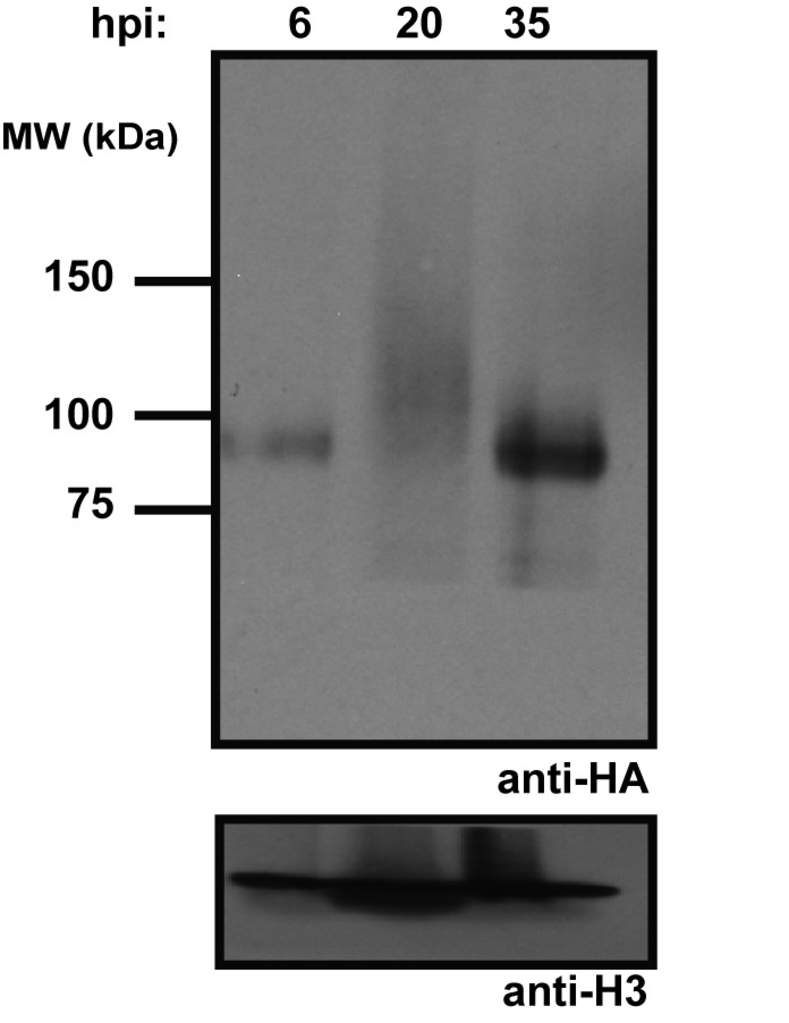

Supplement: S11 Fig — AP2-EXP expression was tracked throughout the IDC by harvesting protein from highly synchronous asexual blood stage parasites followed by a western blot against the 2xHA tag. Histone H3 was used as a loading control. (TIF) [file ppat.1010887.s022.tif]

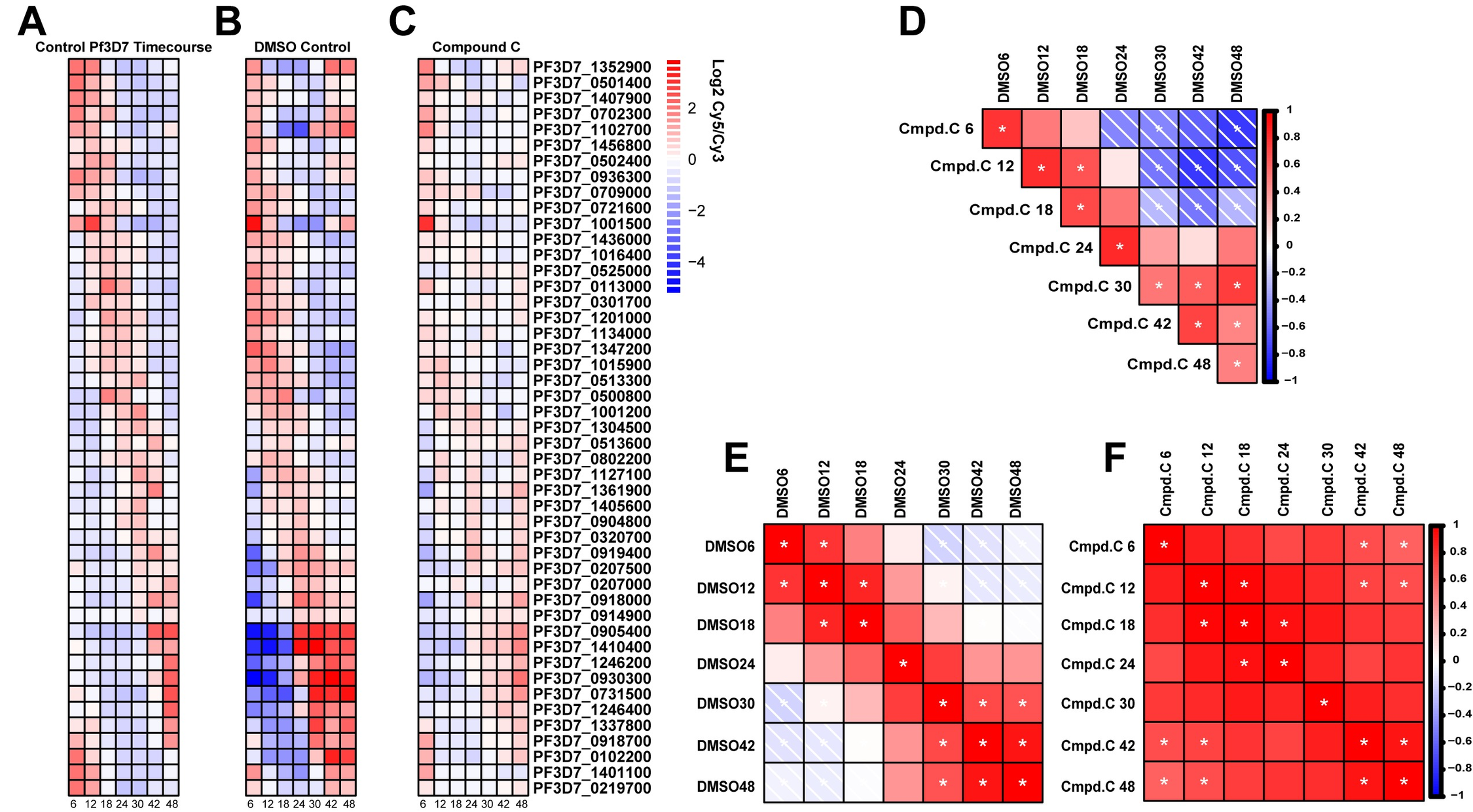

Supplement: S12 Fig — A) DNA microarray data from [51] for a set of highly periodic control genes expressed in the IDC. B-C) The same set of highly periodic control genes as in panel A were plotted for the DMSO control and 12μM Compound C spiked parasites in order to compare parasite staging between the two experiments. D) Correlogram depicting the Spearman Correlation value between control gene expression for DMSO (Panel B) and Compound C (Panel C) samples. A * indicates p value < 0.05. E-F) Correlogram depicting the Spearman Correlation value between the total transcriptome of DMSO control and Compound C dosed parasites. A * indicates p value < 0.05. In contrast to the minimal perturbation observed in either dataset relative to the control gene set, the total transcriptome is significantly altered in the Compound C treated parasites compared to the DMSO control. (TIF) [file ppat.1010887.s023.tif]

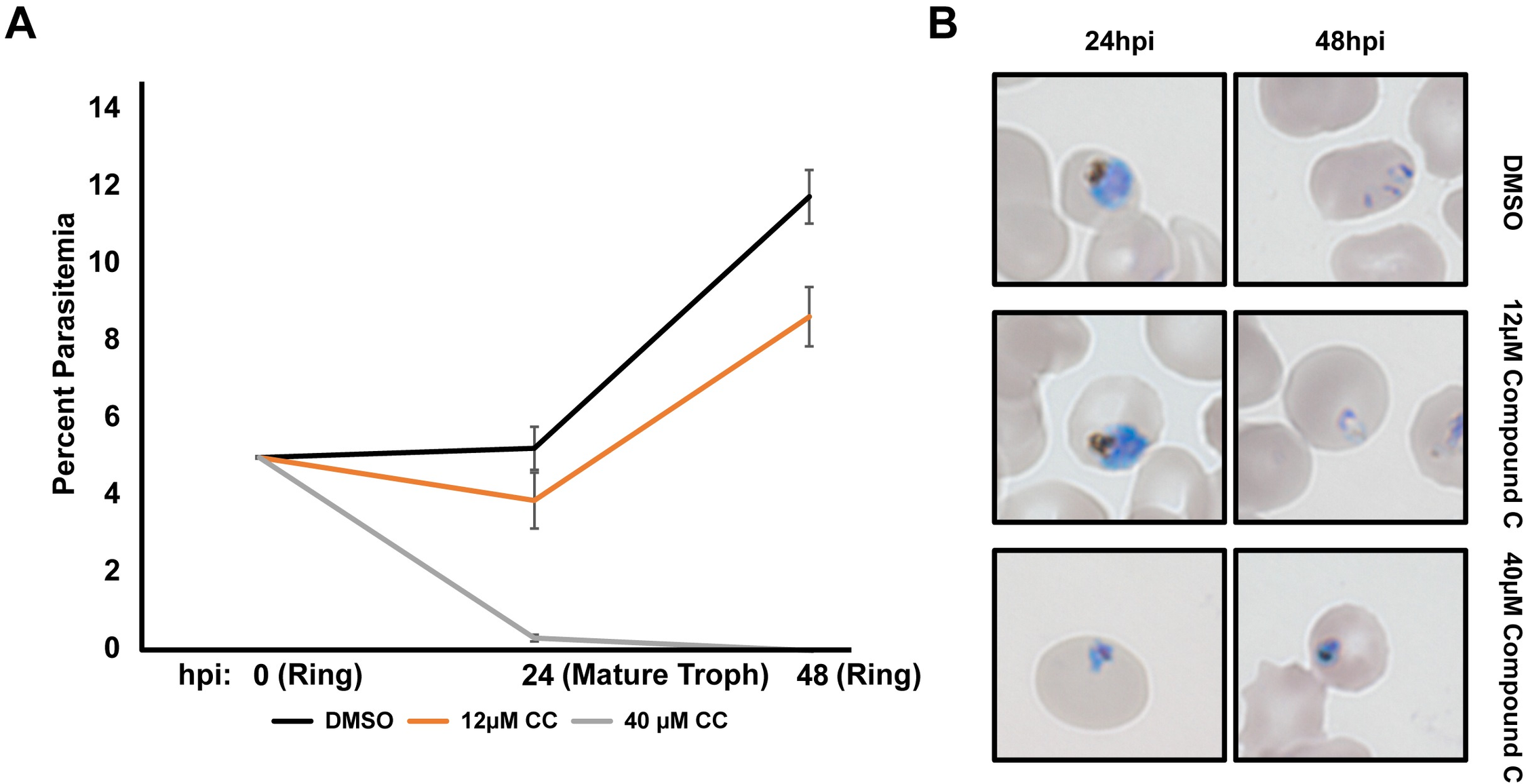

Supplement: S13 Fig — A) Highly synchronous asexual blood stage Pf3D7 parasites were spiked with DMSO vehicle control, 12μM Compound C, or 40μM Compound C. Each growth assay was performed in biological triplicate. Error bars represent standard deviation of the mean. B) Representative images of each parasite population (DMSO, 12μM Compound C, 40μM Compound C) at 24 and 48 hpi. (TIF) [file ppat.1010887.s024.tif]

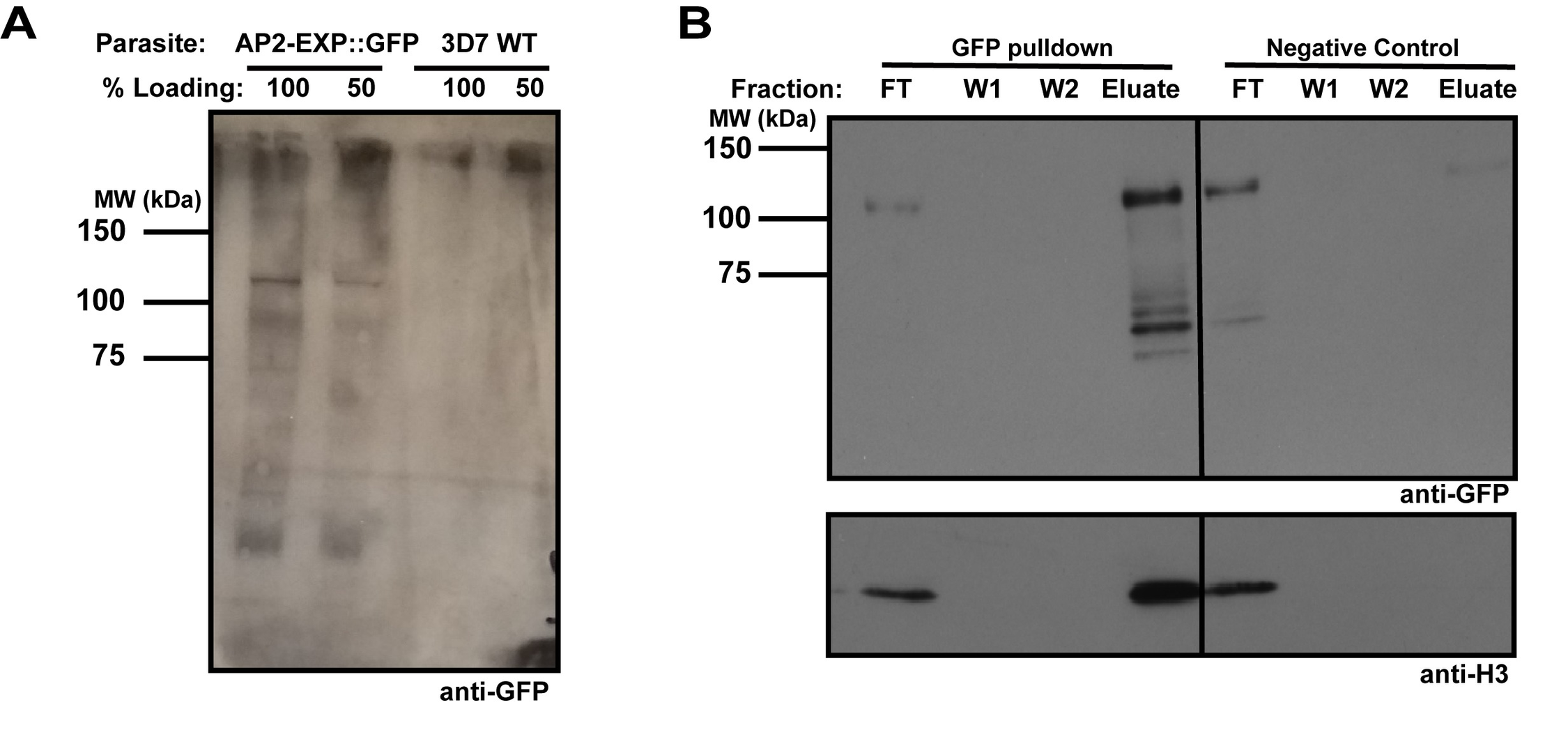

Supplement: S14 Fig — A) Crosslinked nuclear material was blotted after sonication to ensure recovery of the full-length AP2-EXP protein during chromatin immunoprecipitation. Full length AP2-EXP is recovered, indicating that the protocol is suitable to analyze AP2-EXP DNA binding in vivo. Crosslinked nuclear material from the wildtype Pf3D7 parental parasite line was used as a negative control. B) Anti-GFP beads were used to pull down GFP tagged AP2-EXP from AP2-EXP::GFP. Flowthrough (FT), Wash (W1 and W2) and Eluate fractions were analyzed by western blot. The presence of AP2-EXP and Histone H3 in the Eluate lane indicates that AP2-EXP interacts with chromatin in the nucleus. The non-immune negative control beads do not enrich AP2-EXP or Histone H3 in the eluate. (TIF) [file ppat.1010887.s025.tif]

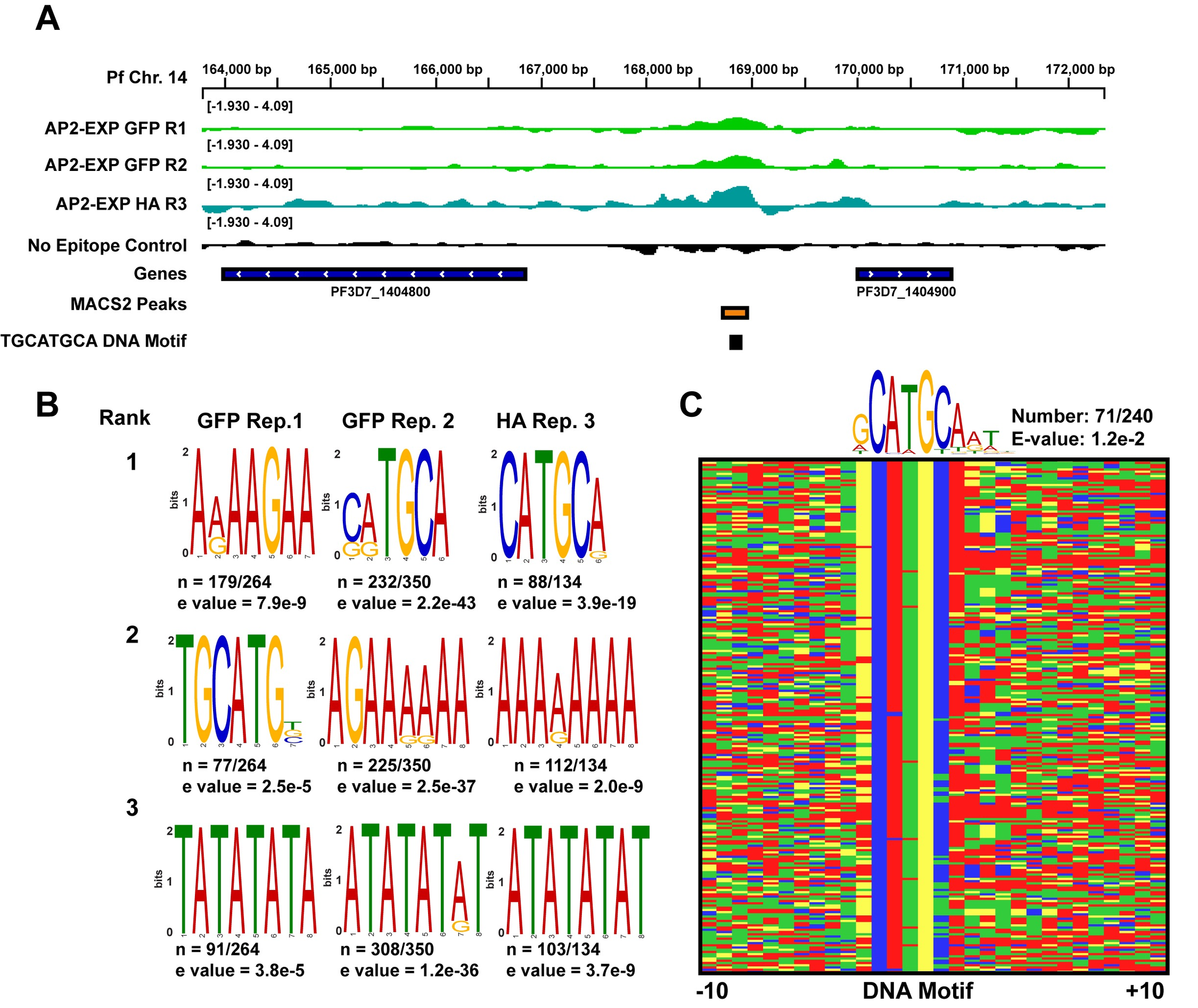

Supplement: S15 Fig — A) Log2 immunoprecipitate/Input ChIP-seq data from each replicate (2x AP2-EXP::GFP and 1xAP2-EXP::HA) of AP2-EXP ChIP-seq visualized by IGV at a representative DNA locus. The location of a conserved MACS2 called peak of occupancy and the TGCATGCA DNA motif is indicated by the bottom tracks. The no epitope control lane is the coverage resulting from a no-epitope control ChIP-seq done using the anti-GFP antibody. B) The top three ranked DNA motifs present within peaks of occupancy for each ChIP-seq replicate as determined by DREME [89]. The core DNA motif CATGCA is overrepresented within each individual replicate. C) The top overrepresented DNA motif within AP2-EXP peaks of occupancy conserved in 2/3 replicates of ChIP-seq as determined by DREME [89] plotted at the primary DNA sequence level. DNA sequences were sorted from highest to lowest degree of motif conservation using FIMO [90]. (TIF) [file ppat.1010887.s026.tif]

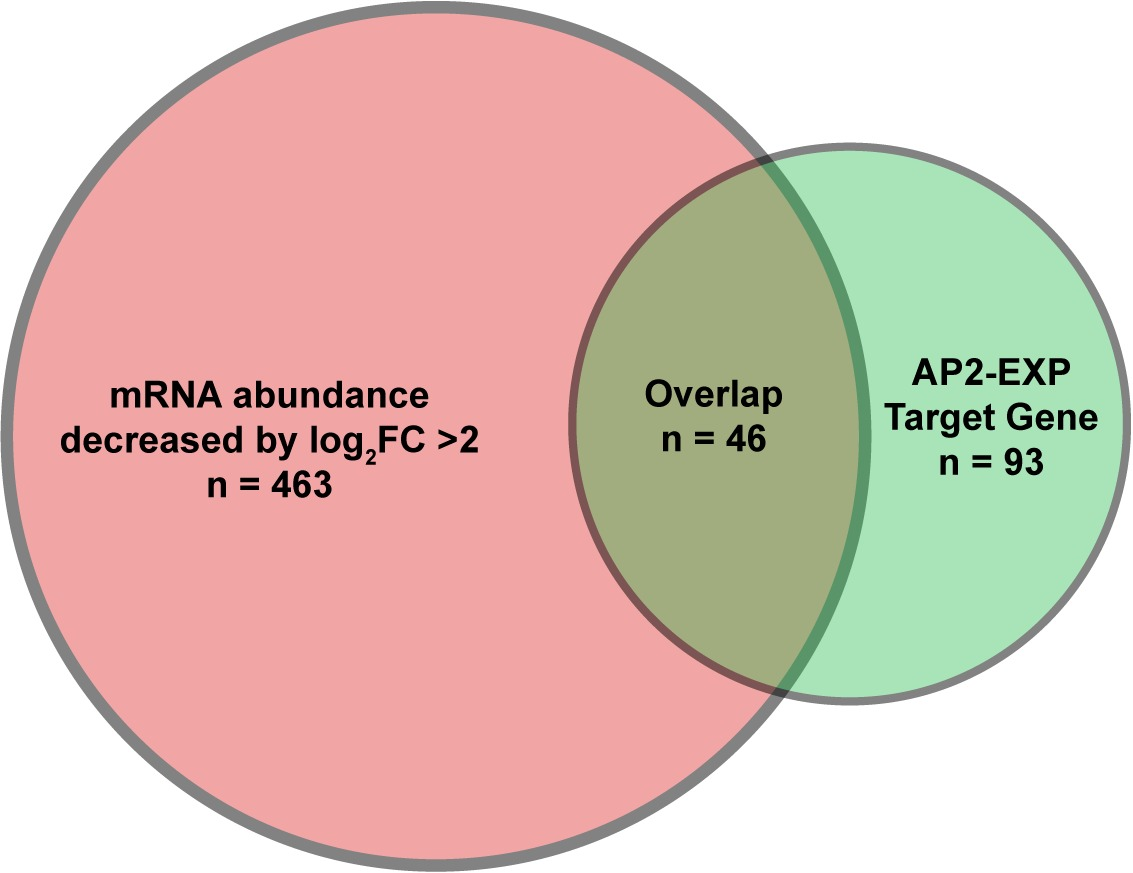

Supplement: S16 Fig — The total overlap between AP2-EXP gene targets detected in the Compound C RNA time course and global decrease in transcript abundance at 24–30 hpi by log2 fold change >2. (TIF) [file ppat.1010887.s027.tif]

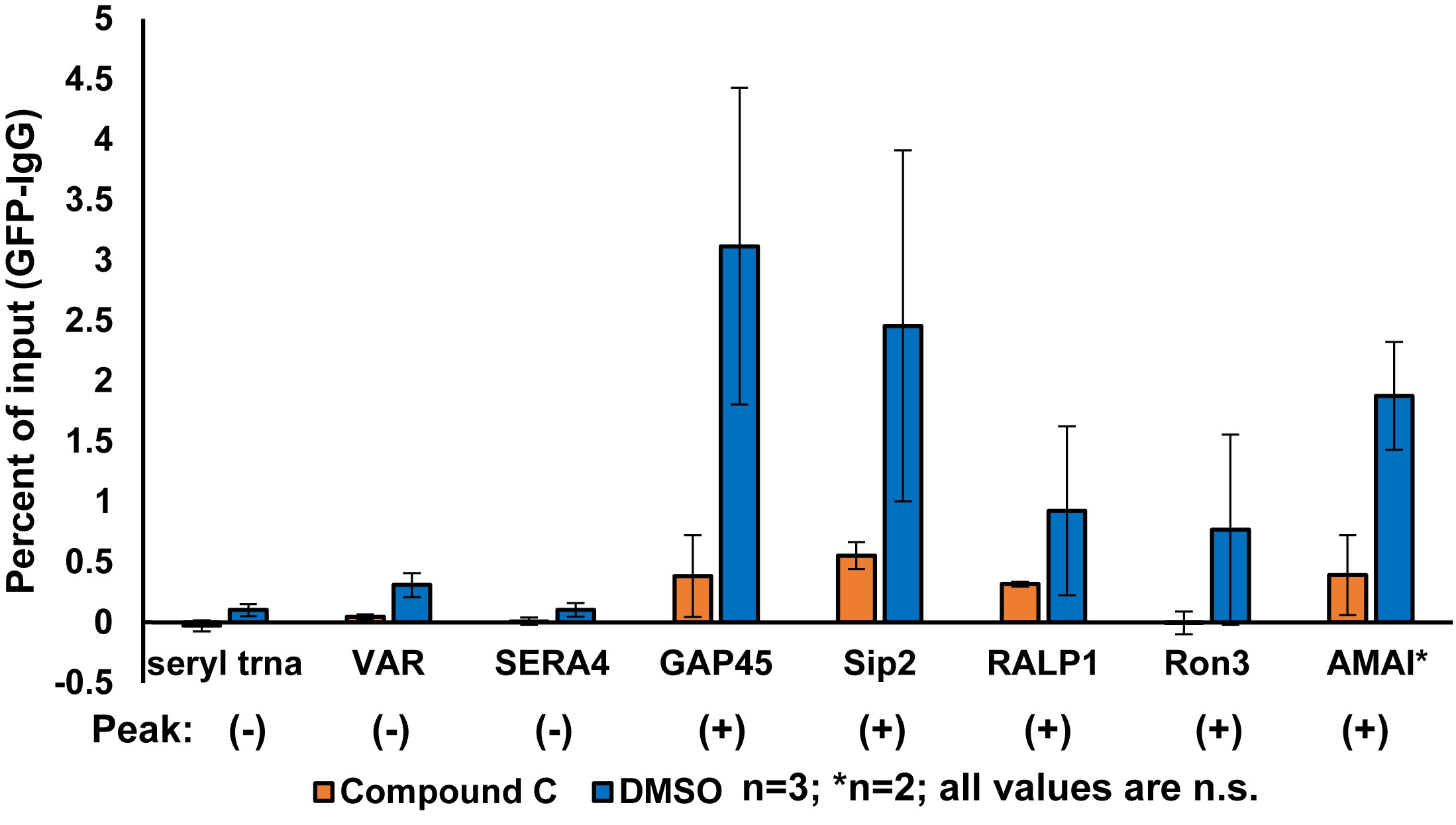

Supplement: S17 Fig — AP2-EXP::GFP parasites were spiked with 40μM Compound C or DMSO vehicle control at 30 hpi for two hours. ChIP samples were collected for each population using either anti-GFP or negative control IgG antibodies. The percent of input was determined by RT-qPCR. The presence or absence of an AP2-EXP peak of occupancy at each DNA locus based on ChIP-seq is indicated by a (+) or (-), respectively. Each assay was done in triplicate with the exception of AMAI, where n = 2. Statistical significance was assessed using an unpaired t-test. All results are non-significant (p-value >0.05). (TIF) [file ppat.1010887.s028.tif]

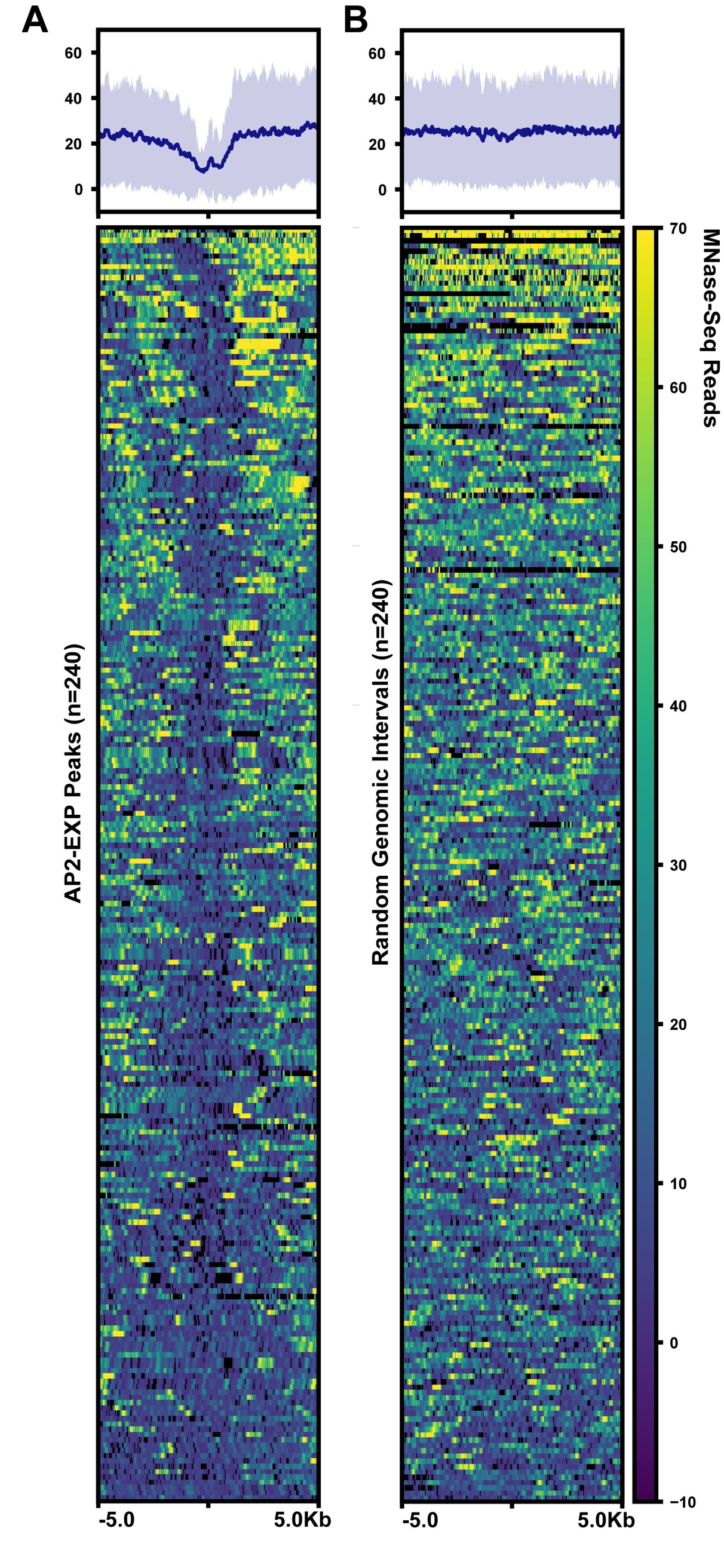

Supplement: S18 Fig — Mnase-seq data [93] was plotted against DNA binding sites conserved in 2/3 replicates of AP2-EXP ChIP-seq (A) or random genomic intervals of equal length from the same chromosome as the original peak (B). (TIF) [file ppat.1010887.s029.tif]

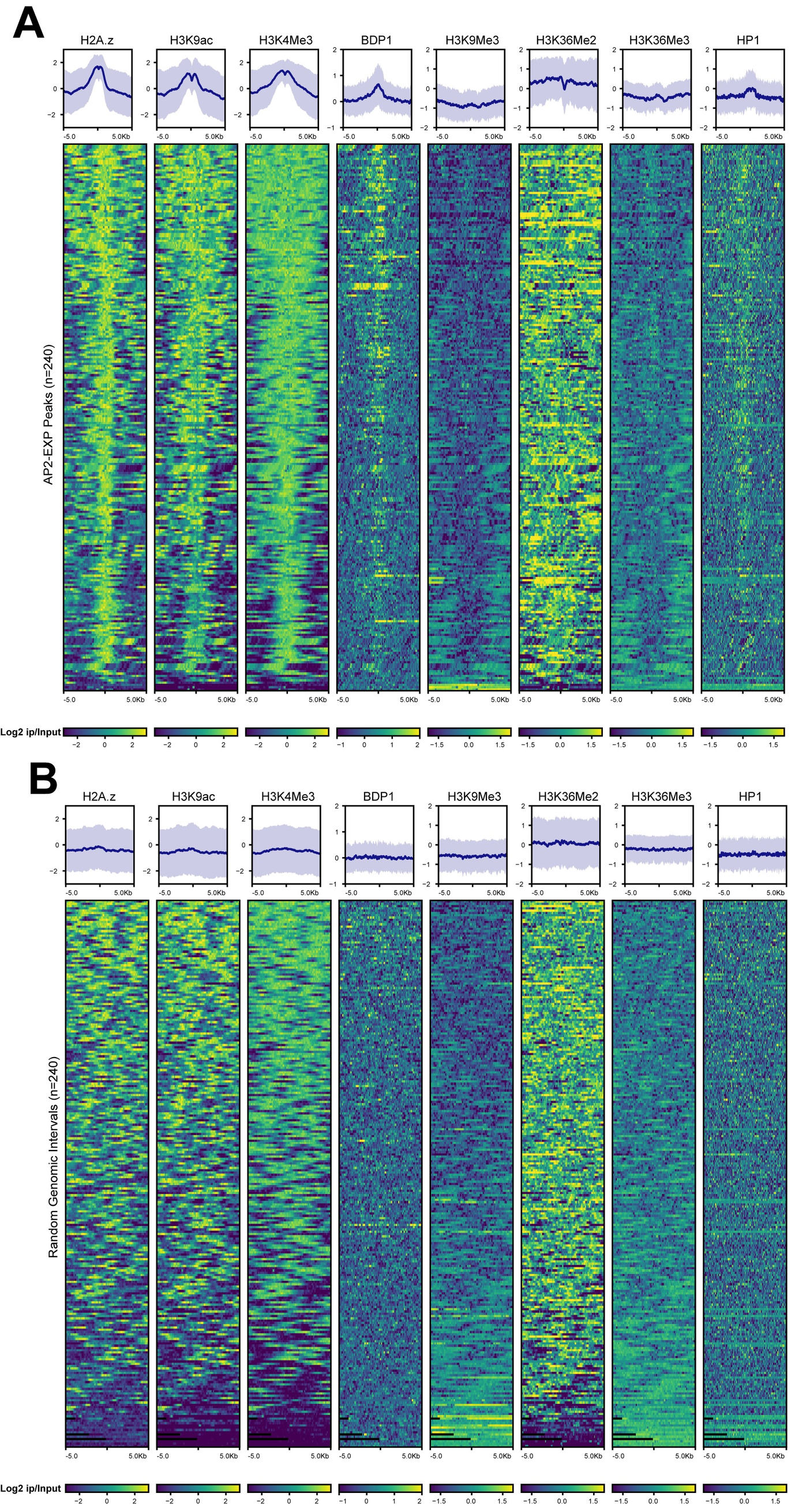

Supplement: S19 Fig — The occupancy of histone variant H2A.Z, histone modifications H3K9ac, H3K4me3 [57], H3K9me3, H3K36me2/3 [91], and chromatin readers BDP1 [58] and HP1 [94] were plotted against AP2-EXP peaks of occupancy conserved in 2/3 ChIP-seq replicates (A) or random genomic intervals of the same length (B), taken from the same chromosome on which the AP2-EXP peak originally occurred. (TIF) [file ppat.1010887.s030.tif]

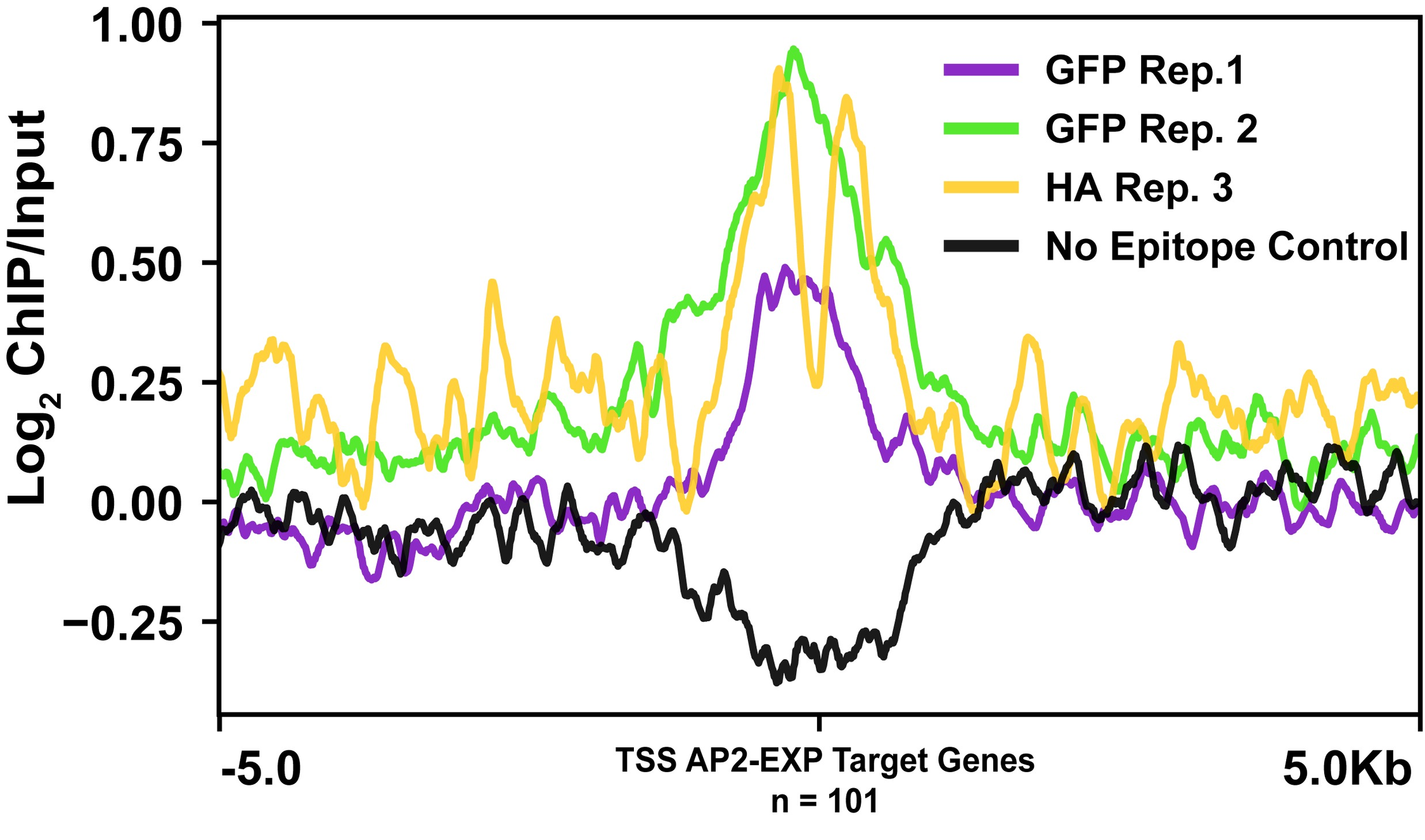

Supplement: S20 Fig — Log2 immunoprecipitate (ChIP)/Input ChIP-seq coverage for each replicate of AP2-EXP ChIP-seq and the no epitope control was plotted against the TSS [59] of each target gene conserved in 2/3 ChIP-seq replicates. (TIF) [file ppat.1010887.s031.tif]

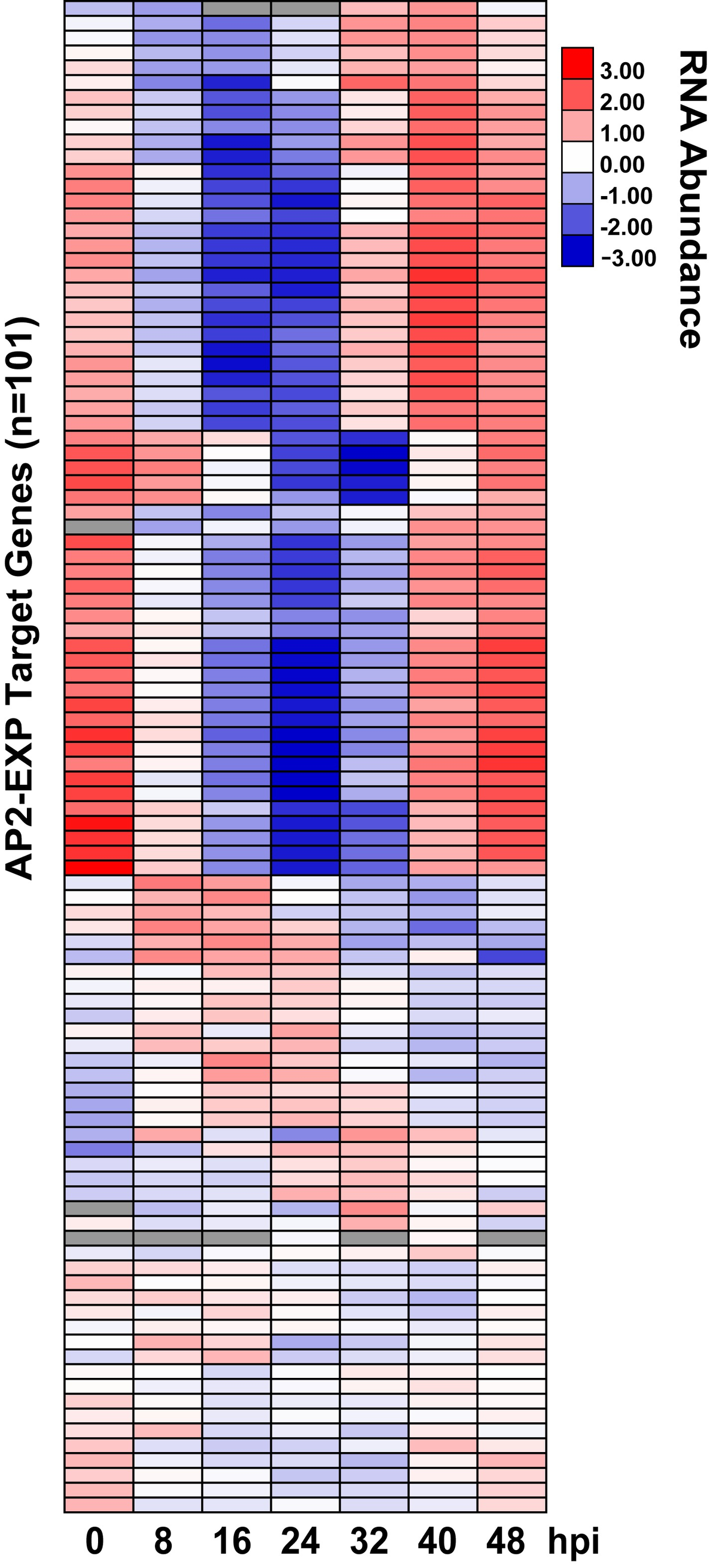

Supplement: S21 Fig — AP2-EXP target genes were determined by ChIP-seq and their transcript abundance data during the 48-hour IDC was plotted using data from Chappell et al [59]. (TIF) [file ppat.1010887.s032.tif]

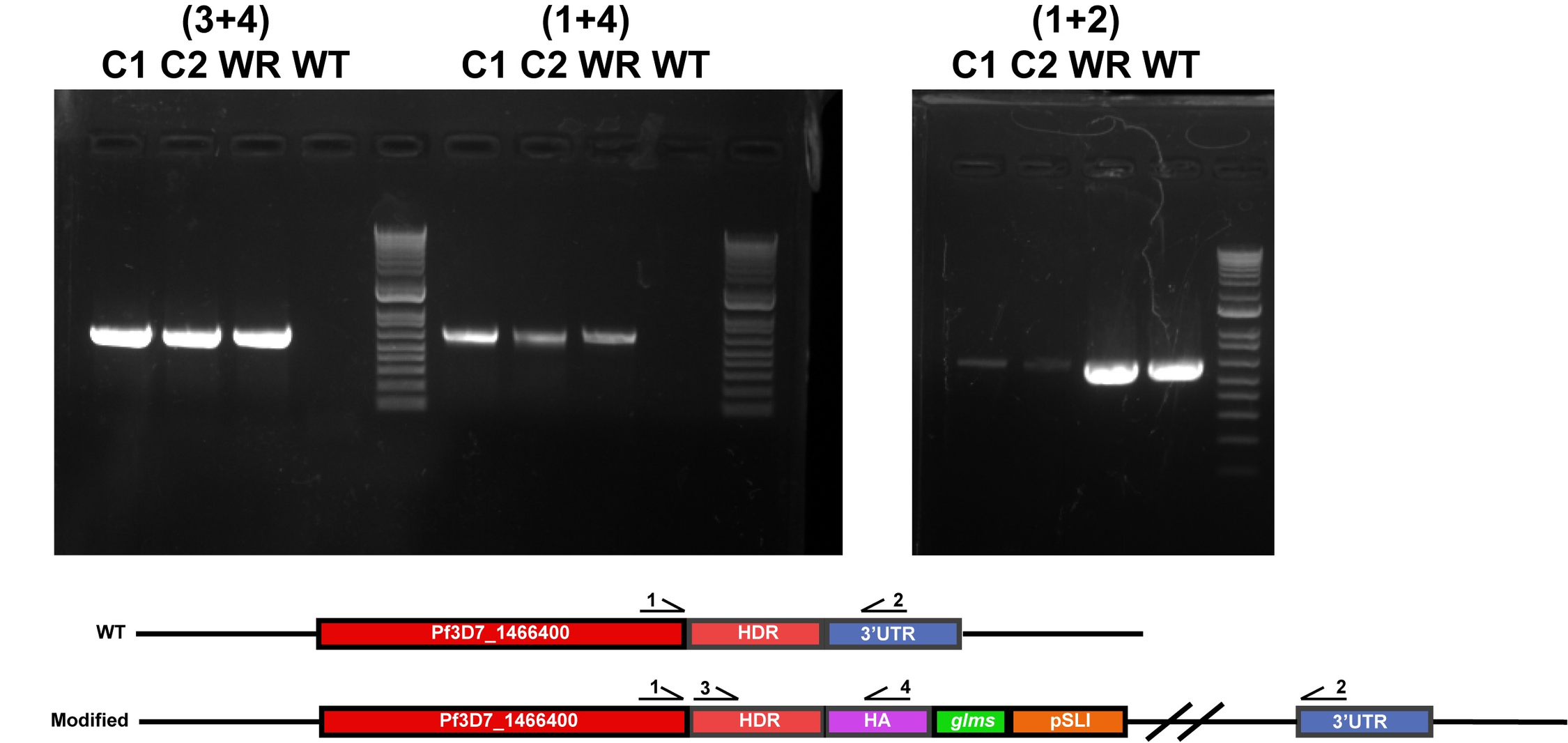

Supplement: S22 Fig — Wildtype Pf3D7 P. falciparum parasites were transfected to endogenously tag the AP2-EXP DNA locus with an inducible glms ribozyme and HA epitope tag. Successful integration to create AP2-EXP::glms::HA by single crossover homologous recombination was confirmed by genotyping PCR. C1 and C2 represent clonal populations selected for integration. WR represents a parasite population selected for the plasmid but not for integration. WT represents Pf3D7 wild type control parasite gDNA. (TIF) [file ppat.1010887.s033.tif]

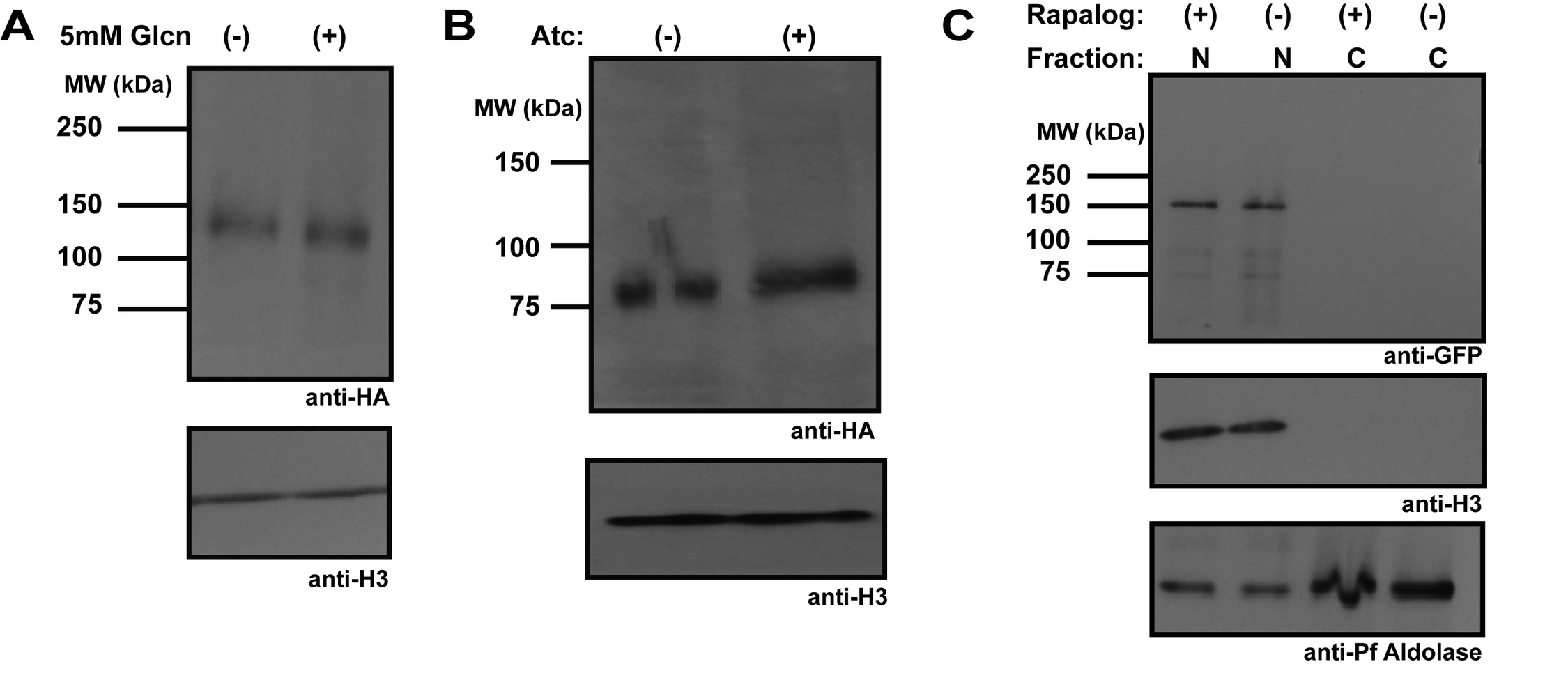

Supplement: S23 Fig — A) To assess genetic knockdown of AP2-EXP by glms ribozyme tag in the parasite line AP2-EXP::glms::HA, highly synchronous parasites were spiked with 5mM glucosamine or vehicle control for 72 hours and AP2-EXP quantity was determined by anti-HA western blot. Histone H3 was used as a loading control. Glucosamine treatment did not impact the amount of AP2-EXP protein present. B) Genetic knockdown of AP2-EXP by the TetR:DOZI mRNA repression module was assessed in the parasite line AP2-EXP::HA by washing anhydrotetracycline (aTc) out of the media for 120 hours. AP2-EXP quantity was determined by anti-HA western blot, and Histone H3 was used as a loading control. Removal of aTc from the media did not impact the amount of AP2-EXP protein present. C) Genetic knockdown of AP2-EXP via protein mislocalization was assessed for the parasite line AP2-EXP::GFP. 250nM rapalog was added to the media for 48 hours and AP2-EXP protein localization was assessed by ant-GFP western blot. Histone H3 and Aldolase were used as nuclear and cytosolic markers, respectively. The addition of rapalog did not cause any detectable mislocalization of AP2-EXP from the nucleus to the cytosol. N indicates the nuclear protein fraction, and C indicates the cytosolic fraction. (TIF) [file ppat.1010887.s034.tif]

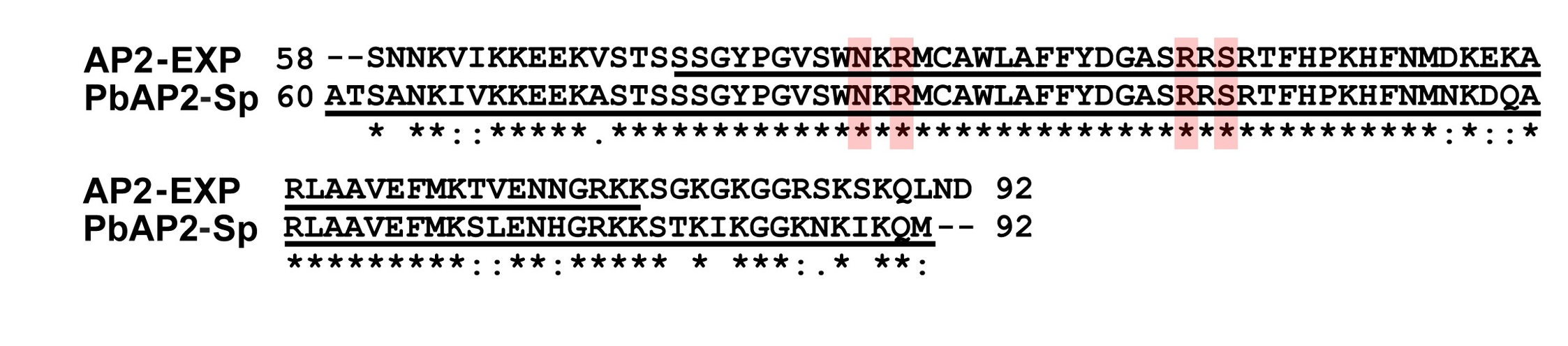

Supplement: S24 Fig — A sequence alignment between the AP2 domain of AP2-EXP and PbAP2-Sp. The four amino acids which make base specific contacts with DNA in AP2-EXP are highlighted in red [40]. The domain boundaries of recombinant PbAP2-Sp [30] and AP2-EXP [12] are underlined. (TIF) [file ppat.1010887.s035.tif]

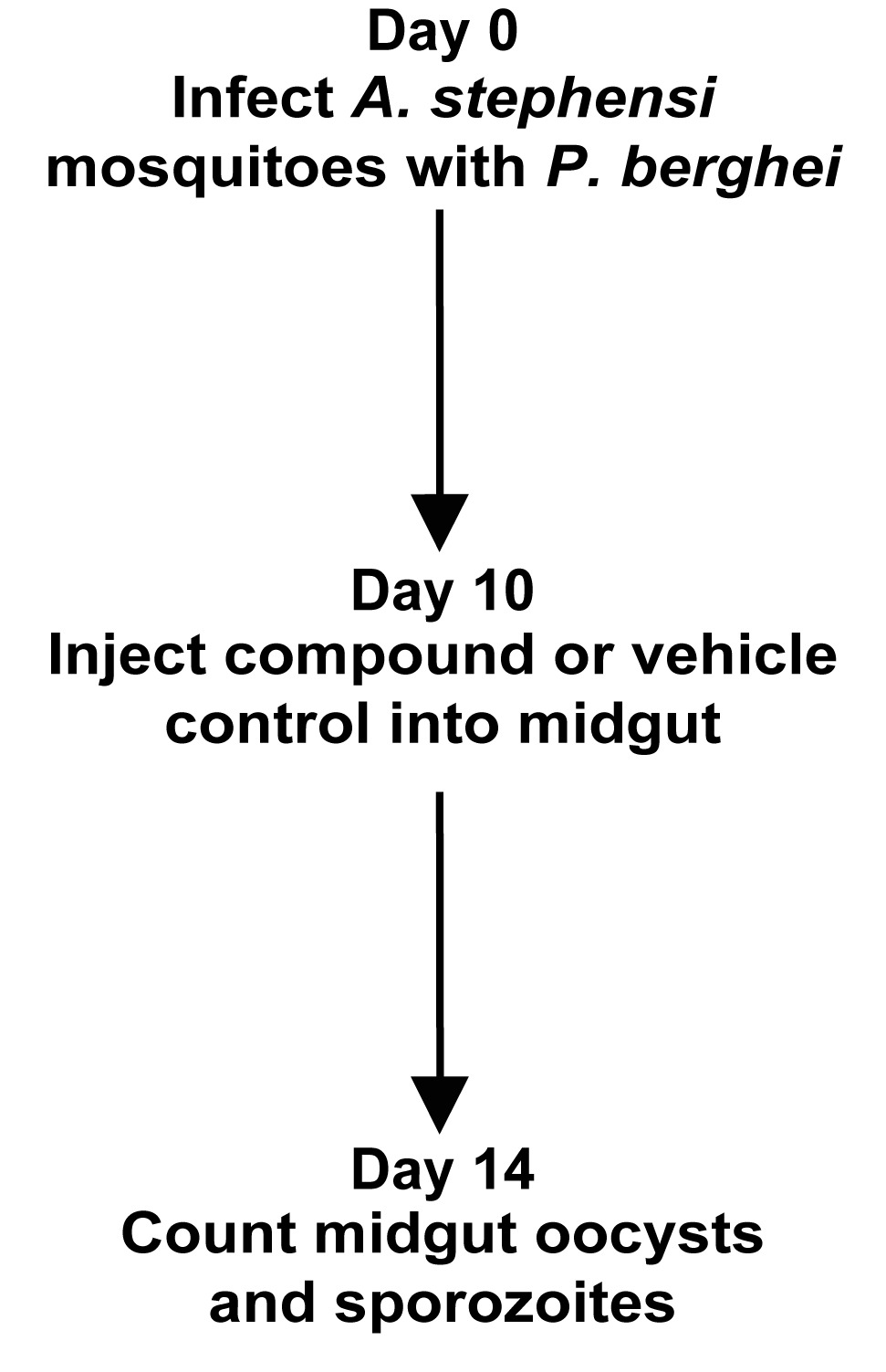

Supplement: S25 Fig — A. stephensi mosquitoes were infected with Plasmodium berghei parasites. On day 10 post infection, mosquito midguts were injected with Compounds B, C, F, or DMSO vehicle control. On day 14 post infection mosquitoes were dissected to count oocysts and midgut sporozoites. For Compound C and DMSO vehicle control, each experiment was performed in duplicate. Compounds B and F phenotyping were performed as a single experiment. (TIF) [file ppat.1010887.s036.tif]
