# Supplementary material for: Inhibitors of ApiAP2 protein DNA binding exhibit multistage activity against Plasmodium parasites
Source: PLoS Pathog. 2022 Oct 12;18(10):e1010887. doi: 10.1371/journal.ppat.1010887 (PMC9591056; doi:10.1371/journal.ppat.1010887)
Supplement: Attachment — Submitted filename: PLOS Pathogens reviews - response to reviewer final.docx [file ppat.1010887.s037.docx]

**General responses to reviewers**

We thank each of the reviewers for their input. We have worked to address all comments and this has resulted in a stronger manuscript which more clearly communicates our findings.

Concerns were raised over the assertion that ApiAP2 proteins are inhibited *in vivo*. We have modified the manuscript title, the discussion, and interpretation of results to reflect this. We have also modified the manuscript to address the potential disconnect between inhibition of AP2 domain DNA binding *in vitro* and the phenotypes observed *in vivo*, while still comprehensively providing evidence that altered transcript abundances are observed in the presence of putative ApiAP2 competitors.

Each reviewer has expressed concerns that the asexual blood stage phenotyping assays for Compounds B, C, and H against *P. falciparum* are not intuitive or adequately described in the original manuscript. We have added new comprehensive phenotyping experiments for Compounds B, C and H wherein the parasitemia and growth stages were recorded at 6-hour intervals during the IDC. Additionally, the Compound C growth assays in the original manuscript (Figure 3C and 3D) have been replaced with a new experiment (Figure 3F) which we believe more clearly represents our findings. These new results address the following parameters: the dosage time for Compound C has been changed from variable to fixed length, and the end point phenotyping assay has been changed from reinvasion by counting rings to a ratio of rings to stalled trophozoites, which better reflects the asexual blood stage phenotype of Compound C.

Several reviewer comments indicated that the measured IC50 values for Compounds A-I appear qualitatively different from the IC50 plots in Figure S3. We have reanalyzed the original underlying data and realized that the IC50 values we originally reported for Compounds B, D, E, F, H, and I were indeed incorrect. We report updated, more precise values for each compound IC50 as well as a detailed explanation of IC50 calculation in the revised manuscript. Considering the reanalyzed results, we find that ApiAP2 inhibiting Compounds A, B, and C (3 of the 4 AP2 domain inhibiting compounds) have the lowest IC50 values among the screened molecules, which should alleviate concerns raised regarding the lack of correlation between ApiAP2 inhibition and IC50 value for the compounds investigated.

The reviewers also commented on the discordance between the similar IC50 values of Compounds B-1,2,3, and 4 despite the different outcomes for each in DNA gel shift inhibition assays. In order to increase confidence that effective antimalarials with non-ApiAP2 targets can be accurately differentiated by the *in vitro* DNA gel shift assay, we have added a new DNA gel shift assay wherein antimalarials with known, non-DNA binding disruption related modes of action (pyrimethamine, chloroquine, and artemisinin) are unable to inhibit an AP2-EXP DNA gel shift in Figure S5G. We expand further on our interpretation of these results in the point-by-point responses and in the revised manuscript.

An additional concern which was raised is the disconnect between systems tested in the manuscript (*e.g.,* after demonstrating that Compounds B and C inhibit AP2-EXP, they are tested against *P. berghei* for life cycle progression inhibition *via* inhibition of the AP2-EXP orthologue PbAP2-Sp). To address this, we have added a new DNA gel shift wherein PbAP2-Sp is inhibited by Compounds B and C but not by the negative control Compound F. This outcome is consistent with our life cycle progression inhibition result, where Compounds B and C have a greater effect on *P. berghei* mosquito stage development than Compound F (Figure 5A).

Please note, in the point by point responses we have used red font for our replies.

Reviewer's Responses to Questions

**Part I - Summary**
**Please use this section to discuss strengths/weaknesses of study, novelty/significance, general execution and scholarship.**

Reviewer #1: The manuscript describes in silico identification of a new class of antimalarial compounds targeting the parasite-specific family of transcription factors and their validation both in vivo and in vitro. It identifies some highly promising hits, shows they are binding the proteins of interest and confirms their ability to inhibit parasite growth. As a follow-up the authors use one of these compounds to investigate its putative target -AP2-EXP, and the role it plays in the parasite maturation and gene expression regulation.


The work is very well designed, clearly presented and data-rich. It contains a very thorough validation of the antiparasitic activity of the studied compounds (authors use two parasite species, multiple life stages etc.). There is no doubt it will be of high interest to the parasitology community, especially as the apiAP2 proteins the authors are targeting are present not only in Plasmodium, but also in other Apicomplexa parasites affecting humans and livestock. The manuscript has therefore the potential to pave way for the discovery of a new class of broad-spectrum anti-protozoan compounds which would have significant medical and veterinary implications.

We thank the reviewer for their positive summary and feedback.

Reviewer #2: This report from Russell and colleagues describes the identification and characterization of small molecule inhibitors of AP2 DNA binding factors in the human malaria parasite Plasmodium falciparum. Using an in silico screen, they identified a number of compounds that are predicted to bind the DNA interaction domain of AP2-EXP, the only AP2 for which the structure is published. The in silico hits are tested for in vitro binding of the protein and in vivo activity against cultured parasites from which, the prioritized compound is then subjected to further study to validate the mechanism of action in parasites. This study adds to the growing body of knowledge on the role of AP2 factors in Plasmodium developmental regulation, and provides preliminary validation that they may be suitable targets for antimalarial drug development.

We thank the reviewer for their positive summary and feedback.

Reviewer #3: In this manuscript titled "Identification of antimalarial compounds that inhibit Apicomplexan AP2 transcription factor proteins in the human malaria parasite Plasmodium falciparum", Russell and colleagues used in silico docking on the AP2-EXP DNA binding domain to identify potential inhibitors of ApiAP2-DNA interactions. They identified 9 compounds (A-H) based on 7 hits as potential inhibitors of ApiAP2 DNA binding that inhibited asexual parasite replication with IC50s between 11 and 170µM. Four of these (A-C,I) were able to block the interaction between AP2-EXP and DNA in vitro. In addition to AP2-EXP, the effect on DNA binding of 3 additional ApiAP2 domains were also determined. A,B,C and I inhibited DNA binding of 50%, 100%, 75% and 100% of the ApiAP2 domains tested. Two hits (A,I) were subsequently excluded because they also blocked DNA binding of either SOX2 HMGB (A) or ERF1 AP2 (I) with DNA. Four analogs of B (B1-4) were also tested for inhibition of AP2-EXP DNA binding in vitro, with B-1 and B-4 able in interfere with binding. Reported IC50 and inhibition of DNA binding did not correlate for B and its analogs. Compound C was found to arrest parasite growth at any point up to mature schizonts at 40µM. Some parasite were able to recover from exposure during the first 24h of the cycle.

Treatment with Compound C at 12µM resulted in a substantial disruption in transcription of which 10% where found to be potentially regulated by AP2-EXP based on upstream binding as determined by AP2-EXP ChIP-seq. The effect of compounds B, C and F, on oocyst maturation as also tested by injecting these into mosquitoes 10 days after blood-feeding and assaying oocyst number and oocyst spz number 4 days later. Compound C did not effect oocyst number but blocked spz formation at 18µM while compound B resulted in complete loss of oocysts at 40µM.

Unfortunately, the presented results are unable to convincingly support the assertion in the title that these compounds function by inhibiting DNA-binding in the parasite even at the micromolar concentrations used.

We thank the reviewer for the summary and constructive feedback. We have worked to address the major concerns brought forth in this summary by rewriting our interpretation of the results to address the possibility of a combination of off target effects and AP2 domain protein inhibition. These are highlighted in detail below.

**Part II – Major Issues: Key Experiments Required for Acceptance**
**Please use this section to detail the key new experiments or modifications of existing experiments that should be** absolutely required to validate study conclusions.

Generally, there should be no more than 3 such required experiments or major modifications for a "Major Revision" recommendation. If more than 3 experiments are necessary to validate the study conclusions, then you are encouraged to recommend "Reject".

Reviewer #1: In my opinion there are no major experiments required to complete the manuscript. There are, however, a few things that should be taken onto account while interpreting the data (see below)

We thank the reviewer for their positive feedback. Our efforts to address additional concerns brought up by the other reviewers are highlighted below.

Reviewer #2: Figure 3B (Lines 194-201). What was the rationale for administering all compounds at 40uM? They all have different IC50s. Is it surprising to the authors that Compound B was apparently able to completely inhibit parasite development at half the IC50 concentration reported in Figure 1?

1) We used 40uM of compound for each growth assay reported in the original Figure 3B because higher concentrations of some compounds would have resulted in the addition of >1% DMSO into the culture media. This is not desirable, because higher concentrations of DMSO cause an asexual blood stage growth defect. Therefore, we chose to add the highest concentration of drug X IC50 possible for the phenotyping assay. In our IC50 growth inhibition assay we used a vehicle control to account for the effect of DMSO, since the readout is parasite DNA content rather than growth phenotype. For this reason, we continued to use 40uM of each compound in our redesigned growth phenotype assay in the new Figures 3A-E of the revised manuscript.

2) We have noted that several reviewer comments point out that the IC50 curves for Compounds B, E, and F look qualitatively different from the calculated IC50. We therefore used the same underlying data to re-calculate the IC50 values and discovered that the IC50 values for these compounds were indeed different from what was originally reported. We thank you for encouraging us to re-assess these data. Upon re-analysis, the IC50 for Compound B was calculated to be 17uM, so 40uM is >2x the IC50. The original value reported was due to a recordkeeping error and we apologize for this oversight. The updated IC50 values are in Figure 1A and Figure S3 of the revised manuscript.

My interpretation of the compound spike-in/wash assays is that a longer treatment time is more effective at inhibition of parasite growth. If I understand the experimental setup correctly, the treatments from Figure 3C and 3D with maximal parasite inhibition were all exposed to compound for >20 hr, compared to only 8 hours for either the 14 hpi wash out in 3C or the 46 hpi spike-in in 3D. You cannot conclude that the 30-46hpi is most sensitive to Compound C treatment without consistent time periods of exposure to the compound.

We agree with the reviewer that in Figure 3C of the original manuscript, the highest drug efficacy correlates with the longest treatment time. However, in the original Figure 3D, the compound has been spiked in only at the time point indicated. In other words, for the 38 hours post invasion timepoint the drug was able to arrest parasite growth completely with only 10 hours of treatment time. This is less drug exposure time than the 14, 22, or 30hpi washout time points in Figure 3C. None of these treatments were able to completely arrest parasites despite having less treatment time.

To address these shortcomings, we have replaced Figure 3 panels C and D with a new drug phenotyping assay in Figure 3F and Figure S8 of the revised manuscript. In this assay, we have added drug for fixed intervals of time (8 hours at a time, throughout the 48-hour asexual cycle) and quantified both reinvasion and the number of stalled trophozoites at 54 hours post invasion. We hope that you will agree that this provides a more comprehensive view of the drug’s timing for maximum efficacy and is free of the potential bias introduced by longer treatment times. Our new findings indicate that any interval of Compound C exposure increases the ratio of stalled trophozoites to reinvaded rings at 54 hpi, but the 22-30 hpi exposure interval has a maximum effect on growth.

A comparison is made between the targets of AP2-EXP identified in this study by ChIP-seq and the targets of a different AP2, AP2-EXP2 (PF3D7_0611200) from Shang et al 2022. It is unclear, and not discussed by the authors how this is a meaningful comparison, particularly since their target list did not correlate with the previously published transcriptomic analysis of truncated AP2-EXP from Martins et al 2017. Based on sequence comparison or structural modeling, would AP2-EXP2 be predicted to be a target of Compounds B or C?

In the original manuscript, we compared the DNA occupancy of AP2-EXP, not AP2-EXP2, from the Shang *et al*. 2022 dataset to our own AP2-EXP ChIP dataset. We apologize if this was not clear.

Reviewers 2 and 3 have both pointed out the possibility of comparing more AP2 domain protein target genes with our DNA microarray time course dataset in order to draw conclusions about the possibility of multi-ApiAP2 inhibition. To address this, we have used the newly available ApiAP2 ChIP-seq data from Shang *et al*. 2022 (PMID: 35288749) to make comparisons using the target genes of each of the 13 ApiAP2 proteins analyzed in Shang *et al*. 2022. This data has been added to Table S3 and is discussed in lines 424-446. Overall, we find that many ApiAP2 protein target genes are affected by Compound C, but the greatest proportion of genes affected are targets of AP2-EXP and AP2-I.

Reviewer #3: 1. While compounds B and C are able to block DNA binding in vitro and kill parasites, the evidence that they reduce DNA-binding in the parasite and whether this is their mechanism of action is circumstantial at best.

To the contrary, compounds B, B-1, B-2, B-3 and B-4 have very closely related structures and kill parasite with near identical IC50s (ref 38) but B-2 and B-3 do not block DNA binding/

We thank the reviewer for pointing out the disconnect between the calculated IC50 values and *in vitro* AP2 domain inhibition. We have now recalculated the IC50 values of Compounds A-I and discovered that several of the IC50 values (Compounds B, D, E, F, H, and I) were incorrectly reported. We apologize for this oversight. Compounds A, B, and C have the lowest IC50 values in our growth inhibition assays and represent 3 of the 4 AP2 domain competing compounds identified in the initial tests. Therefore, these results are now consistent.

In order to address whether Compound C decreases AP2-EXP DNA binding, we completed a ChIP-qPCR experiment (Figure S17) and found that AP2-EXP abundance is decreased at 5 out of 5 loci tested, albeit without statistical significance. We have changed the manuscript title to better reflect the results reported. Furthermore, we have significantly reworded our discussion regarding the putative anti-DNA binding activity of the compounds in the revised manuscript to indicate that DNA binding inhibition takes place *in vitro* (*e.g.*, lines 364-369). We have also added references to a recent series of publications wherein a DNA binding interface blocking molecule was refined to decrease off-target effects, thereby demonstrating the potential to build from the results described in this manuscript (lines 378-382).

We sourced Compounds B-1,2,3, and 4 from GlaxoSmithKline due to their high similarity to Compound B and the fact that they are known antimalarials. Our intent was to determine whether we could describe any determinants of blocking DNA binding by AP2 domains *in vitro,* rather than to assert that all molecules similar to Compound B must target ApiAP2 proteins *in vivo*. We have modified lines 189-194 and 390-405 to reflect additional interpretations of these results, including the potential for Compound B analogues to target different, untested AP2 domains, or to have other completely off-target effects.

Furthermore, we have added new DNA gel shifts with the recombinant purified AP2-EXP AP2 domain (Figure S5G, lines 400, 170-177) wherein the known antimalarial drugs pyrimethamine, chloroquine, and artemisinin have been added to a competitive EMSA. None of these antimalarials compete AP2-EXP DNA binding. We added this result to provide evidence that highly effective antimalarial drugs do not generally compete for AP2-EXP in vitro, supporting our claim that the DNA gel-shift assay is capable of differentiating the putative ApiAP2 competing compounds from antimalarials with different modes of action.

2. It makes little sense to ascribe the effect of B & C to inhibition of AP2-EXP.
Despite the fact that compounds B and C inhibited DNA binding for 100% and 75% of the ApiAP2 domains tested
Extrapolating this observation to the 40 ApiAP2 domains across 27 proteins would suggest binding of most ApiAP2s would be impaired.
This is consistent with the observation that AP2-EXP binding was only found in 10% of down-regulated genes (Fig 19)
Also, since animals dont have AP2 domains, wouldn't a pan-ApiAP2 inhibitor be even better?

We agree with the reviewer that from a drug development perspective, a pan-ApiAP2 inhibitor may be the best outcome. We have modified lines 108-110, 154, 321, 377-382, 403-405, and 424-454 to reflect this.

We choose to focus on providing evidence that AP2-EXP is minimally one of the targets of Compound C because it is the protein domain that the compound was originally docked against computationally, therefore providing evidence that AP2-EXP is impacted by Compound C is our most direct means of assessing whether the drug is having the predicted effect.

We provided a table in the original manuscript (Table S3) comparing all gene targets of the AP2 domain proteins inhibited by Compound C *in vitro* (AP2-EXP, SIP2 and AP2-I) to transcript abundances in the presence of Compound C. However, to address the possibility of multi or pan-ApiAP2 inhibition, which has been raised in multiple comments, we have now added a tab to Table S3 in which we have compared the gene targets of each of the 13 ApiAP2 proteins recently analyzed using ChIP-seq by Shang et al 2022 (PMID: 35288749) to the transcript dysregulation caused by Compound C. We have also commented on the likelihood of multi ApiAP2 inhibition in the discussion (lines 424-454).

3. The stated IC50 of 80µM for B is wrong. It neither agrees with curve in S3B, not the activities of the B1-4 analogs. The graph indicates a value around 10µM, closer to the reported value in Gamo et al. (0.93µM).
The IC50 of B1-4 should be determined alongside B to allow comparison. The reported values for E (170 vs ~350), F(40 vs 70µM) also look off.

We thank the reviewer for noting the incorrect reporting of IC50 values. These values were incorrectly stated due to a clerical error. We have updated all IC50 values after re-analyzing them using the original underlying data (Figure 1A, Figure S3).

4. The phenotyping experiments (Figure 3, S13) need to be carefully redone with detailed methods and more readouts than re-invasion at a specific time-point (see comments below)

We agree with this suggested improvement to our data, and we have noted that each reviewer asked for clarification or improvement on the assays in Figure 3C and D and Figure S13 of the original manuscript.

To address this, we re-ran the phenotyping assay depicted in S13 using 6-hour interval time points as in the original Figure S14 for Compounds B, C, and H. We have recorded both the parasitemia and the parasite morphology at each time point. The new data are reported in Figure 3 A-E.

For Figure 3C and D, we used reinvasion as our endpoint assay because we established that the phenotype for Compound C is trophozoite stage arrest, which prevents reinvasion. To address concerns about this assay, we have changed the assay to a fixed-length drug dosage of 8-hour intervals in the IDC. We have recorded both the number of arrested trophozoites and the reinvasion rate at 54 hpi in order to fully report the phenotype for Compound C. The data are reported in the revised manuscript in Figure 3F and Figure S8A, B and are consistent with our original reported results.

5. Figure legends largely fail to indicate the number of biological replicates, the statistical basis of the error bars, and when results are significantly different.

In the original manuscript, we have indicated the number of biological replicates for each assay in the figure captions where applicable. We have also indicated the nature of the error bars or asterisks indicating statistical significance where applicable in each figure caption.

To address concerns about displaying significance and sample size more effectively, we have added statistical tests for data presented in Figure 3F (Compound C fixed interval exposure phenotyping, Mann Whitney U-test), in Figure S17 (Compound C spike in vs. DMSO control AP2-EXP ChIP-qPCR, unpaired t-test) and Figure 5B, C (Compound B, C and F mosquito stage *P. berghei* phenotyping experiment, Mann Whitney U-test). In Figure 4A we have added an explanation of the indicator for statistical significance to the figure itself. In Figure 3A-D, Figure 3F, and Figure 5B, C we have added an indicator of the sample size to the figure.

6. Results that are being directly compared in the text are often separated between the main and supplementary figures, making it very difficult for the reader to evaluate the support for these claims.

We thank the reviewer for pointing this out. We generated a large body of data and worked to ensure that as much as possible was represented in the main figures. In order to present our main findings more fully, we have now added extensive parasite phenotyping data for Compounds B, C, and F to Main Figure 3.

**Part III – Minor Issues: Editorial and Data Presentation Modifications**
**Please use this section for editorial suggestions as well as relatively minor modifications of existing data that would enhance clarity.**

Reviewer #1: 1. While all the described compounds have been designed against the AP2-EXP DNA-binding domain, it is clear that this is not their only target. Importantly there seems to be little correlation between the compound’s ability to displace AP2-EXP- bound DNA in vitro and its antiparasitic activity (IC50). The fact that the modifications that abolish the AP2 binding activity of a compound across different domains (eg B-3 variant) may even improve its lethality (fig 2) suggests that these additional targets may be proteins other than apiAP2s. While investigation of these additional targets would be very laborious and is probably out of the scope of this paper, their exitance should be acknowledged.

We agree with the reviewer’s comments. These concerns align well with those raised by reviewer 3 in the major comments section. We have addressed the apparent lack of correlation between the IC50 values reported and the AP2 domain inhibiting activity by re-analyzing the data underlying the growth inhibition plots. Fortunately, this led us to determine that 3 out of 4 of the compounds which can compete AP2 domains (A, B, C) have the lowest IC50 values out of the nine original compounds tested (Lines 149-150).

We agree that the similar IC50 values for each of the Compound B derivatives, despite differing activity against AP2 domains, indicates that they are likely to have additional targets. We have added additional interpretation of the results (lines 189-194, 400-405) to reflect this. In addition, we have added a new DNA gel shift experiment (Figure S5G) wherein we demonstrate that some highly effective antimalarials do not compete DNA binding by AP2-EXP. We interpret this to indicate that the competitive EMSAs can distinguish between antimalarials that do or do not affect AP2 domains *in vitro*, regardless of their parasite killing efficacy. Finally, we have added an explicit acknowledgement stating that we cannot rule out additional off-target effects (Lines 321, 377).

2. Given the above, the use of compound C to investigate the function of AP2-EXP may suffer from potential off-target effects. While I agree that the temporal correlation between the protein expression and compound activity is suggestive, it is not sufficient to claim that the resulting phenotype is the result of AP2-EXP inhibition. An interesting way to test this would be to use the compound against the asexual stages of the rodent malaria parasite. They were shown to require Ap2-exp for sporozoite development but not for asexual replication and therefore should not be affected by compound C at this stage.

We agree with the reviewer that testing against the *P. berghei* asexual blood stage could provide details about specificity for AP2-Sp for Compound C. However, we expect that Compound C will kill asexual blood stage *P. berghei* due to its activity against additional AP2 domains in vitro, including AP2-I D3, which has an orthologue encoded on the essential *P. berghei* ApiAP2 protein PBANKA_1205900. We choose to run our development assay against the mosquito stage of *P. berghei* because it was our most direct means of assessing whether PbAP2-Sp could minimally be one of the targets of Compounds B and C. We have modified our explanation of the timing of action of Compounds B and C against *P. falciparum* to indicate that any protein target in addition to AP2-EXP could be responsible for the IDC arrest phenotype (Lines 223, 452).

3. Authors show that the compound C is active against both P.berghei and P.falciparum even if the sequences of AP2-EXP proteins and their function differ between the two parasites. Are the key residues interacting with the compound conserved between the two species? If so can the prediction be made regarding the activity of the compound against eg. P.vivax ortholog?

We thank the reviewer for this suggestion. In order to fully address the capacity of Compounds B and C to compete DNA binding by the *P. berghei* AP2-Sp AP2 domain, we sourced *P. berghei* genomic DNA, cloned the AP2 domain, and overexpressed and purified PbAP2-Sp to use in a gel-shift inhibition assay that we now include in our revised manuscript. We found that Compounds B and C do compete PbAP2-Sp DNA binding, while Compound F (the negative control) does not (Figure 5A). We also have added a sequence alignment for PbAP2-Sp and AP2-EXP in the Supplement (Figure S24), wherein we have indicated the location of the four amino acids that make DNA base specific contacts in the AP2-EXP crystal structure. Between these largely homologous AP2 domains, all 4 DNA-contacting amino acids are conserved. Therefore, we believe that this new result corroborates our *P. berghei* development assay and provides initial evidence that these compounds are potentially useful against additional *Plasmodium* species (Lines 343-358).

4. The previous work, the Western blot in fig S26 and the function of AP2-EXP, all indicate nuclear localisation. In figure S11 however the protein appears to be mostly cytoplasmic. Is that an artefact of the added tag? Or a result of protein processing? Was it observed also with the HA-tagged variant or is it unique for GFP?

By our assessment, the GFP signal appears disperse but concentrated in the nucleus. We have relied on the western blots to further narrow down localization. By western blot, we have not detected strong evidence of protein processing (FigS10A, S11) and our fractionated western blot indicates that AP2-EXP is exclusively nuclear in localization (Fig S23C). AP2-EXP was detected in the nuclear proteome by Oehring, S. *et al* 2012 (PMID: 23181666) and AP2-EXP was detected in the nucleus of 9/12 proteome samples but in the cytosol of only 1/12 samples by Batugedara, G. *et al* 2020 (PMID: 32017676), further increasing our confidence that it is nuclear in localization.

5. Previous work by. Martins et al. identified Ap2-EXP as a regulator of clonally variant gene families in Plasmodium. The authors don’t mention whether this finding was confirmed using their transcriptome data. Were these genes included in the analysis?

We agree with the authors that this comparison is important to make. In the original manuscript, we discuss the correlation between our ChIP-seq based prediction of target genes for AP2-EXP and the genes dysregulated in the Martins *et al*. study (Lines 416-422). We also provided a comparison between our results and Martins *et al*. in Table S4 of the original manuscript. Overall, the overlap was very minimal with only one gene in common between the Martins dataset and our list of AP2-EXP target genes. Due to the nature of the AP2-EXP genetic manipulation in the Martins *et al*. publication (overexpression of just the AP2 domain coding region) it is difficult to predict what the phenotype correlation between AP2-EXP AP2 domain overexpression and AP2-EXP chemical knockdown may be.

Also – minor technical details:

6. In figure S8 3 it is unclear what are the compounds on the X-axis labelled B,K,N are (probably a leftover from an old naming system).

We thank the reviewer for pointing this out and we have fixed the names. This was a carryover from our original naming system.

7. Table S3 would benefit from inclusion of the gene names or product descriptions to facilitate exploration

We agree that this enhances the use of Table S3 and have added gene ID descriptions to each sheet.

Reviewer #2: Figure 2. Compound B-1 has the lowest IC50 against blood stage parasites but shows poor inhibition of AP2-EXP DNA binding (compared to Compound B and B-4). While I acknowledge that there may be many explanations for differences in in vitro and in vivo activity of a molecule, the 1000-fold difference for Compound C-1 is striking. The authors should discuss this finding in a little more detail. Do they expect B-1 to bind more efficiently to another AP2 domain, or is it possible that it has other, non AP2 targets?

We agree with the reviewer, as well as reviewers 1 and 3 that this discrepancy should be further addressed in the manuscript. We have described our changes to the manuscript to address this in our response to reviewer 3’s major point 1, as well as reviewer 1’s minor point 1.

More detail is needed on how the growth inhibition assays were performed and IC50s calculated. The reference #63 that is provided in the Materials and Methods section does not exist. (The title is from a patent with different authors than the ones included in the reference).

We thank the reviewer for noticing this issue. We have modified the reference to properly cite the IC50 inhibition assay used (Bacon, D. *et al.* 2007, PMID: 17220424). We have also provided additional details regarding the IC50 value calculations to the methods section (Lines 565-578).

Figure 3C and 3D.
The way this data is presented is not very intuitive. The authors should consider including a simple experimental overview of how compound was spiked in/washed out for each time point.

We acknowledge that all three reviewers have pointed out that the assay in Figure 3 C and D is not intuitively presented. To address this, we have run a new microscopy phenotyping experiment described in our responses to reviewer 2’s major point 2, and reviewer 3’s major point 4 that now replaces the original data presented in Figures 3C & 3D.

Approximately 50% of dysregulated genes after Compound C treatment were predicted targets of AP2-EXP based on the ChIP-seq assay. Are there any commonalities in the other half of dysregulated genes? In the Discussion, the authors mention that a proportion of the dysregulated genes are predicted AP-I targets but many of these are also AP2-EXP targets. Did the authors perform motif analysis on the upstream regions of the other dysregulated genes that are not targets of either AP2-I or AP2-EXP to see if there are enriched motifs that may help to predict if Compound C inhibits any other AP2 factors? Is it likely the Compound C binds multiple AP2 domains?

We have worked to address this point by providing a new comparison of ApiAP2 ChIP-seq data from Shang *et al* 2022 (PMID: 35288749) to TableS3 in which we compare each of the newly described ApiAP2 target genes to our DNA microarray time course. We have used these new comparisons to speculate on the potential for multi ApiAP2 competition *in vivo* in lines (424-446). Additionally, we did mine the 50% of AP2-EXP gene targets that are not affected by Compound C for potential regulatory DNA motifs using the DREME suite software, but we did not find a predicted cis-regulatory motif that differs from the TGCATGCA DNA motif which is enriched in the AP2-EXP target genes as a whole.

Reviewer #3: OTHER COMMENTS & QUESTIONS:

The introduction is completely missing background/context about previous efforts to develop small molecules that interfere with protein-DNA binding for therapeutic use in other systems.

We thank the reviewer for pointing out this relevant omission. We have added a brief overview of the current state of the art in small molecule-based inhibition of DNA binding proteins, with a specific focus on nuclear hormone receptor and DNA motif blocking agents. We have also added one of the limited number of examples of attempts to drug the DNA:protein interface currently in the literature by highlighting efforts to interfere with the transcription factor STAT3 (lines 93-102, 373-382).

All figures: Please ensure text in figures/table is selectable so readers can easily copy drug IDs, etc

We apologize that the text was not selectable in this review copy. We will work with the editorial staff to rectify this.

FIG 1B:
Why is binding inhibition not quantified?

We used an ECL-based imaging system to visualize the biotin labeled DNA oligonucleotide after probing with streptavidin HRP. Since the signal output from ECL is not linear, we cannot quantify the exact inhibition. Therefore, we have presented whether DNA binding is abolished, greatly decreased, or remains constant relative to the control.

Fig 1C:
Structural similarities for A-C extend beyond benzaxolone and benzaxolone is clearly no sufficient for inhibition since D doesn't inhibit

We have modified the text (lines 147-149, 188-190) to indicate that Compounds A,B, and C are not the only molecules in our initial group which contain the benzoaxole moiety. We did not intend to state that the benzoaxole moiety alone is able to compete DNA binding, rather, that it emerged from the *in vitro* test as a common feature of 75% of the AP2 domain competing compounds.

Can you make useful inferences about SAR based on CID4541005 being inactive vs CID1365835/CID5750730 ?

We thank the reviewer for this suggestion. We were unable to complete an SAR follow up study at this time. Instead, we opted to take an empirical approach to narrowing down which aspects of an AP2 domain inhibiting compound can change its activity *in vitro* by testing the four closest available derivatives of Compound B (B-1 through B-4).

Also dont all of the structures have multiple planar rings? Worth noting similarities to DNA bases?

We have added this observation to our justification for the ethidium bromide exclusion assay (Lines 179-180). The presence of planar rings analogous to DNA bases is what prompted us to run the exclusion assay to test whether any of the compounds intercalate DNA.

CID for compound C is wrong, should be CID 5750730 (124220 is the TCMDC ID of the docking hit)

We thank the reviewer for noticing this error. We have fixed the reference to the CID.

line 132: Is it clear that binding is competitive?

We agree with the reviewer that the lack of DNA binding observed in a DNA gel-shift does not strictly demonstrate competition has taken place. Instead, we have reworded the activity of AP2 domain inhibiting compounds to indicate that they abolish or prevent DNA binding throughout the manuscript. When we refer to ApiAP2 inhibition, we state that the definitive evidence of inhibition is extrapolated from the *in vitro* EMSAs.

line 150: not blocking SOX2 & ERF1 doesn't demonstrate that B & C are ApiAP2 specific, merely that binding isn't broadly non-specific.

We have changed the wording (Lines 169-170, 384-390) to indicate that our assay only indicates relative specificity compared to these protein domains.

Also why disqualify I for binding ERF1 since it’s also an AP2 domain?

We have disqualified Compound I due to the combination of 1) preventing DNA binding by AtERF1, 2) having the highest magnitude of DNA intercalation ability, and 3) presenting the least effective IC50 among the Compounds A-I.

The EtBr displacement assay is not sufficient that these compounds do not bind DNA by themselves.
EtBr displacement assays were done at 50µM while EMSAs were at 300µM of the inhibitor and DNA used in EtBr assay was only 8bp long, which is less than 1 helical turn.
Moreover, intercalation is only one way that compounds can can bind DNA and block protein DNA interaction (See PMIDs: 22183179, PMID: 30774852)
The gel-shift assay from Figure 5A of PMID 16009327 or Isothermal Titration Calorimetry from <https://pubs.acs.org/doi/10.1021/acs.jmedchem.8b00233> would be more suitable.

We used a DNA intercalation assay described in the Wang *et al.* (<https://doi.org/10.1016/j.matpr.2017.09.190>) reference. We choose to use the TGCATGCA DNA motif in order to assess whether any of the compounds tested specifically interfere with the AP2-EXP DNA binding motif, similar to the sequence specific DNA binding molecules described against various transcription factor motifs (*e.g.*, <https://doi.org/10.1172/JCI92504).> We maintain that the assay is sufficient as reported because the positive control intercalator DRAQ5 can knock down ethidium bromide fluorescence at 1/10^th^ the concentration of the putative ApiAP2 competitors tested in the assay (5uM vs. 50uM). Since the positive control DRAQ5 reduces the EtBr signal, the assay is demonstrated to be sufficient to measure DNA intercalation.

Figure S1B was a bit confusing. Switch B to D that way the analogs similar to TCMDC-124220 are A-C, and the compound similar to TCMDC123924 is D.
It would be interesting that add the predicted binding affinity

We appreciate that the naming convention does not take into account the original docking hit that led to the purchase of each molecule, however, we cannot change the order without having to change the order of the compounds in our *in vitro* gel shift assays. We chose not to change this in order to keep the compounds in alphabetical order in each figure. We have chosen not to add the predicted binding affinities because the criteria (< -5kJ/Mol) for predicted affinity was used as an arbitrary cutoff for the *in vitro* follow up study.

Figure S2: add structures to EC50 plots
Show binding and structure of the 2 unavailable compounds (TCMDC-124220,?
CID for compound C is wrong, should be CID 5750730 (124220 is the TCMDC ID of the docking hit)

We appreciate pointing out the incorrect CID number for Compound C. This has been fixed. We did not add the structure to the EC50 plot in order to avoid redundancy with Figure S2. We have chosen not to provide the docking conformation for TCMDC-124220 since it was never used in the manuscript outside of the initial hit in the assay.

Figure S3:
Does the >2log linear binding curve for B indicates that effect is not via DNA binding?

We do not know of any metric we can use to speculate on the relationship between the IC50 curve for Compound B and its mode of action. While we acknowledge that none of the compounds highlighted in the initial screen have sub-micromolar IC50 values, we have provided preliminary evidence that further optimization is possible (Lines 373-383). We highlight that despite the need for further optimization, we have provided evidence that small molecules that compete DNA binding by AP2 domains *in vitro* are effective against *Plasmodium* parasites.

Combine S5 & S6.

We have combined these figures as suggested.

Why do S4E&F look so different from S6A&E, even in the absence of compounds?

We found that adding DMSO to the gel-shift had an adverse effect on the aesthetics of the SOX2 and AtERF1 DNA gel-shifts. We included a DMSO vehicle lane in S6A and E in order to demonstrate that despite the aesthetic differences between DNA gel shifts, adding DMSO does not change the presence of the shifted DNA band.

The structure of ERF1 AP2 bound to DNA is solved (PDB 1gcc) What are the predicted binding affinities of A-H?

We agree that this is an interesting proposal, however, we do not have the ability to robustly compare the predicted affinities of A-I for AP2ERF1 and AP2-EXP due to the lack of conservation of DNA base contacting residues and the very different binding area presented by the monomeric AP2ERF1 domain vs. the homodimerized AP2-EXP AP2 domain. Instead, we used the empirical observations gained from the DNA gel-shifts using AtERF1.

Movies: Not sure the simulations are useful, given that neither B-2 and B-3 block binding but the simulations predict that B-2 interaction is stable but B-3 is not
also Movie S2 & S5 are switched. S2 shows B-4, S5 shows B1 and Movies S3 and S5 lack the indicators for non-carbon atoms.
But if kept, switching the color is unhelpful but adding the predicted bonds formed between compound and protein might be useful visually (but not worth it if its difficult to do)

We thank the reviewer for noticing that the molecules in movies S2 and S5 were labeled incorrectly. These have now been fixed. We agree that the molecular dynamics simulations do not provide a comprehensive overview of each molecule’s activity in the *in vitro* gel shift tests. However, we have kept the movies in the manuscript because the different outcomes for the Compound B analogues potentially provide a model for the difference in outcomes detected in the gel shift assay. We have modified our interpretation of these results in the discussion (Lines 390-400) to reflect this. We are unable to model a diagram of the predicted contacts without significantly altering the nature of the molecular dynamics simulation that was run.

What was the point of B1-4 if follow-up is done with B?

We used Compounds B-1 to B-4 in order to probe for minor changes to an ApiAP2 inhibiting compound that affect its activity *in vitro*. While we were able to utilize B-1-4 for this purpose, we ultimately determined that Compound B has the most activity against AP2-EXP (Figure 2B) and therefore chose to proceed using Compound B instead of B-1-4.

Figure 3 / Figure S13 / Figure S14
Figure S13, S14 results should be included in Fig 3.
If the goal of this figure is to show that B & C have similar effects on parasites then the same phenotyping experiments need to be done for B as were done for C in S14 and 3C-D.

We agree with the reviewer that these growth assays are more informative when all done using the same method. To address this, we have redone the growth assays for Compounds B and H in the same manner as was done for Compound C. The new phenotyping time courses are incorporated into the new Figure 3A-E.

Figure S13: I could not find the starting parasitemia for this experiment in the methods, but based on S13A, it would have been set up at 8% trophs if parasite are schizonts at 24h post spike-in.
That seems like very high starting parasitemia, when growth is to be maintained for another 72h.
The parasitemia of the DMSO treated fails to increase ( 8% at 24h post spike-in, 7% at 48h post spike-in, 9% at 72h post spike-in)
The figure shows parasitemia at 24, 48, 72h after treatment not just 48 as indicated in the figure title.

We agree with the reviewer that the starting parasitemia was high given our intent to measure reinvasion. To rectify this, we have re-run the phenotyping assays for Compounds B and H to match the time course done for Compound C (Figure 3A-E).

The cell shown in Fig3B for H-treated cultures at 48h-post spike-in is a gametocyte (note the dispersed hemozoin), is this representative?

We agree with the reviewer that the hemozoin in this image is similar to that normally observed in gametocytes, however, we never recorded any evidence that Compound H dosed parasites undergo increased gametocyte commitment. We have updated the Compound H phenotype description in accordance with the new phenotyping time course in Figure 3A-E.

Figure S13/14: Phenotypes dont agree. Figure 13 shows B & C-treated parasite are rapidly lost, while in S14 parasitemia is constant in C-treated cultures.

We apologize for the confusion created by the presentation of data in Figure S13. We have removed S13 in order to instead show the phenotype caused by Compounds B and H using the same assay as Compound C, in Figure 3A-E.

The phenotypes appear to differ in the original manuscript Figure S13/14 because the graph only showed the percentage of the expected life stage as per the diagram in Figure S13A of the original manuscript.

Figure 3C-D, why is the ring parasitemia in the next cycle the only read-out from this experiment? Are parasites killed or arrested?

Based on this comment, we have redone the experiment depicted in Figure 3C-D using a different method. We now treated parasites for 8-hour intervals with Compound C across the IDC, then recorded the percentage of stalled trophozoites and nascent rings in the culture at 54 hpi. We have reported this as a ratio in Figure 3F and added a diagram with the full data in Figure S8.

Line 191: Just because compounds arrest at at similar point doens't mean they are likely to have the same target.
The fact that the inhibition of DNA binding and IC50s dont seem to be correlated (B, B1-4 have very similar IC50s between 0.25-1.1µM), would suggest that inhibition of AP2-EXP binding isn't the mechanism.

We have modified the manuscript to reflect the lack of correlation between IC50 for Compound B-1-4 and the activity against AP2 domains. We have described our changes to the manuscript to address this in our response to reviewer 3’s major point 1, as well as reviewer 1’s minor point 1. As suggested, we have also changed our interpretation of the similar phenotype for Compound B and C to include potential off-target effects (Lines 223, 378).


Line 197: states that parasites fail to progress but based on S13, they are actually disappearing from the culture.

We have addressed this by redoing the assay for Figure 3A-E.

Lines 575-589: unclear, write separate descriptions of the spike-in and wash-out experiments.
Based on this description, adding drug and immediately washing it out (14hpi timepoint), reduces parasitemia by 50% in the next cycle.
Is that correct? If so, it seems surprising that exposure for 8h (22hpi washout) doens't have much more profound effects, than exposure for only a few minutes.
Are the methods possibly incorrect and C was added at 6hpi (as for S14) and the 14hpi represents the effect of 8h drug exposure?

We apologize for the incorrect description. Compound C was added at 6hpi in the washout experiment. Since we have redone the experiment to instead measure parasite progression following fixed intervals of Compound C dosage, this section is no longer in the revised manuscript.

Figure 3C: How do the authors explain that AP2-EXP is not expressed at 18hpi but exposure to C until 14hpi and 22hpi already has substantial impact parasite growth?

Although it has been consistently reported between our results and those of Martins *et al.* (https://doi.org/10.1038/s41598-017-12578-y) that AP2-EXP is maximally expressed in the middle trophozoite stages, the recent ChIP-seq data reported by Shang *et al.* 2022 (PMID: 35288749) indicates that AP2-EXP is already present and binds DNA in the nucleus in the ring stage. Therefore, it is possible that Compound C inhibits AP2-EXP when it is too low in abundance to be detected by western blot, but still present.

Figure 3D: What was the ring-stage parasitemia in cultures when compound C was added at 46hpi? A substantial fraction of the rings at 54hpi might already have been present at 46hpi.

We visually confirmed a lack of detectable ring stage parasites in this assay. However, the assay in Figure 3D is no longer in the revised manuscript as we have changed the blood stage phenotyping assays.

FIGURE 4

Figure 4A, S15D: The correlation matrices between DMSO-treated time-points (DMSO on both x & y axis) needs to also be shown in order for these figure to be interpretable since it is the difference between these correlation matrices that indicates the change.
Minor point: DMSO / compound C-treated are shown on opposite axes in 4A and S15D. It took me a second to realize that the matrix wont change. To avoid this confusion in other readers, it would be best to keep them on the same axis.

We thank the reviewer for pointing out the inconsistency with the x-y labels. We have fixed this in Figure 4A and S12A-F of the revised manuscript.

We have also added the correlation matrices for DMSO vs. DMSO and Compound C vs. Compound C to Figure S12 of the revised manuscript. The correlation between DMSO control time points shows that the transcriptome progresses normally from 6-48hpi, while the correlation between Compound C time points indicates that the transcriptome is not progressing in the same manner. Since these plots cannot show specific differences between the data, we have kept the Compound C vs. DMSO comparison as the correlation plot for Main Figure 4A.

Lines 224-229:
Correlation between DMSO and C-treated timepoints is lower when all transcripts are used than when only ~50 highly periodic genes are used.
The text states that this indicates that there isn't an overall perturbation of the transcriptome.
However, this would make sense if only a small subset of genes exhibited altered expression but S19 shows that at the 24-30h time-point alone, expression of nearly 10% of parasite genes was altered by at least 2x.
Since, 80% of genes are developmentally regulated and the the number of genes with altered expression greatly exceeds the number genes in the control set, it seems that the opposite is the case: the overall transcriptome is more perturbed than highly periodic genes.

We understand the point the reviewer has raised. Comparisons between the highly periodic control genes are commonly used as a means of assessing the quality of *Plasmodium falciparum* transcriptomics datasets.

We have rephrased our interpretation of these results to indicate that the perturbation at the specific 30hpi time point of interest (this is the point where ~10% of genes are altered) is profound, yet overall, the parasite proceeds normally through its predefined developmental transcript cascade. This assessment is supported by our phenotyping assay in Figure S13, where we demonstrate that in the presence of 12uM Compound C the majority of parasites do successfully proceed through one IDC. This is now discussed in lines 258-263.

Figure 4B: Is the x-axis supposed to show the motif distance to the start codon?

The x-axis hash mark is a reference point for the start codon of all AP2-EXP target genes. The median enrichment of AP2-EXP or the no epitope control is shown on the y-axis. Since not every binding site contains a specific DNA motif, no information about the motif is used to make the metaplot. We have included this information in the figure caption and the methods description for generating the metaplots (Lines 749-751).

Figure 4C: The grey color is supposed to indicated "Non Significant" but genes in green are also non-significant.
Also, based on the Table S3, the y-axis shows the -log10(Adjusted P-value), not the -log10(P-value).

We thank the reviewer for noticing this discrepancy and we have changed the y-axis label. We have changed the plot colors to be consistent with the p-value cutoff used for reporting results from Table S3.

Figure S18: Legend states that the Control (-) track shows the coverage but since coverage can't be negative this must also be enrichment when IP-ing from an untagged line.
Which antibody was used, anti-GFP or anti-HA?

In ChIP-seq experiments, it is common for negative enrichment to be observed due to recovering more material in the control input DNA than in the immunoprecipitated DNA fraction. The Control (-) track in Figure S15A of the revised manuscript indicates the coverage in a no-epitope control ChIP-seq experiment from the untagged line. This was done using an anti-GFP antibody, as indicated in the text. We have changed the label to ‘No Epitope Control’ in the revised manuscript.

Full ChIP-seq (bigwig or bedgraph) tracks need to me made available.

We apologize that bigwig files were not available at the time of review. Fastq files were available on SRA at the time of review, and bigwig files are now available in a GEO submission linked to the original SRA submission. Therefore all sequencing data associated with this work are now publicly accessible.

Figure 5: How did the size and morphology of C-treated oocyst differ from DMSO-treated oocysts.

This information was not recorded quantitatively, however, no size difference was noticed between Compound C oocysts and the DMSO control.

PLOS authors have the option to publish the peer review history of their article ([what does this mean?](https://journals.plos.org/plospathogens/s/editorial-and-peer-review-process#loc-peer-review-history)). If published, this will include your full peer review and any attached files.


If you choose “no”, your identity will remain anonymous but your review may still be made public.


Do you want your identity to be public for this peer review? For information about this choice, including consent withdrawal, please see our [Privacy Policy](https://www.plos.org/privacy-policy).

Reviewer #1: No

Reviewer #2: No

Reviewer #3: No

**End of reviews**

**Figure Files:**

While revising your submission, please upload your figure files to the Preflight Analysis and Conversion Engine (PACE) digital diagnostic tool, [https://pacev2.apexcovantage.com](https://pacev2.apexcovantage.com/). PACE helps ensure that figures meet PLOS requirements. To use PACE, you must first register as a user. Then, login and navigate to the UPLOAD tab, where you will find detailed instructions on how to use the tool. If you encounter any issues or have any questions when using PACE, please email us at [figures@plos.org](mailto:figures@plos.org).

Data Requirements:

Please note that, as a condition of publication, PLOS' data policy requires that you make available all data used to draw the conclusions outlined in your manuscript. Data must be deposited in an appropriate repository, included within the body of the manuscript, or uploaded as supporting information. This includes all numerical values that were used to generate graphs, histograms etc.. For an example see here on PLOS Biology: <http://www.plosbiology.org/article/info%3Adoi%2F10.1371%2Fjournal.pbio.1001908#s5>

Reproducibility:

To enhance the reproducibility of your results, we recommend that you deposit your laboratory protocols in [protocols.io](http://protocols.io/), where a protocol can be assigned its own identifier (DOI) such that it can be cited independently in the future. Additionally, PLOS ONE offers an option to publish peer-reviewed clinical study protocols. Read more information on sharing protocols at <https://plos.org/protocols?utm_medium=editorial-email&utm_source=authorletters&utm_campaign=protocols>
